# Supplementary material for: Association between cardiometabolic disease and severe COVID-19: a nationwide case–control study of patients requiring invasive mechanical ventilation
Source: BMJ Open. 2021 Feb 17;11(2):e044486. doi: 10.1136/bmjopen-2020-044486 (PMC7893210; doi:10.1136/bmjopen-2020-044486)
Supplement: Supplementary data [file bmjopen-2020-044486supp001.pdf]

Online Supplementary data

Cardiometabolic factors and risk for severe COVID-19 requiring invasive mechanical ventilation during the Swedish epidemic

Svensson P, Hofmann R, Habel H, Jernberg T, Nordberg P

Table of contents

Supplemental methods – register information 1

eTable 1 – Hypertension definitions 2

eTable 2 - Missing Data in the Study Population 3

eTable 3a-c - Baseline characteristics of the study population by age-group……..4-6

eTable 4 - Baseline characteristics of the study population by sex 7

eTable 5 - Baseline characteristics of the study population by region of birth...……..8

eTable 6 - Odds ratios for Covid-19 requiring ICU-admission by cardiometabolic factors and other comorbidities (n=1,417 cases and n=14,170 control subjects)...……..9

Supplemental figure S1 - Directed Acyclic Graph……..10

Supplemental figure S2 - Exclusion flowchart ……..11

References……..12

Supplemental methods – register information

The Population Register
The Population Register is managed by Statistics Sweden and includes information on deaths, emigration and immigration for the entire Swedish population. All residents are assigned a unique personal identity number that can be used for linkage of different data resources including several national health registers of high quality.

The longitudinal integrated database for health insurance and labor market studies (LISA)
LISA is managed by Statistics Sweden and includes annual measurements on several socioeconomic and sociodemographic variables, including income, education and country of birth.

The Swedish Patient Register
Swedish Patient Register is managed by the National Board of Health and Welfare and covers inpatient care since 1964- (nationwide since 1987) and non-primary outpatient care since 2001. The register is nationwide with a near complete coverage during the study period.

The register is regulated by the Health Care Data Register Act (1998:543; Lag om hälsodataregister) and the Patient Register ordinance (2001:707; Förordning om patientregister hos Socialstyrelsen). It is mandatory for all physicians, private and publicly funded, to deliver data to the Patient Register. Data from the Patient Register are subjugated to the Health and Medical Services Act (1982:763; Hälso och sjukvårdslag) and the Patient Data Act (2008:355; Patientdatalag). [1]

The Swedish Prescribed Drug Register
The Swedish Prescribed Drug Register is managed by the National Board of Health and Welfare and started on July 1, 2005. The register covers all drugs except over-the-counter medication (which is not covered at all) and medications administered at hospitals (which is only covered to some extent in the Prescribed Drug Register and completely covered through the National Patient Register in some counties).

National Board of Health and Welfare register data is protected by strict confidentiality but can be made available for research after a special review after which data can be provided to Swedish researchers according to standard legal provisions and procedures. Of special importance to the regulation of Swedish medical research and health care is also the Public Access to Information and Secrecy Act (2009:400, Offentlighets- och sekretesslagen). [1]

Svensson P, *et al. BMJ Open* 2022; 11:e044486. doi: 10.1136/bmjopen-2020-044486

eTable 1 – Hypertension definition.

Anti-hypertensive drugs	ATC-codes	Exclusion diagnosis (ICD-10)
Diuretics	C03A, C03D, C03E	
Beta-blockers *	C07A, C07F	Angina pectoris (I208, I209)<br>Atrial fibrillation (I48)<br>MI (I21, I22)<br>Heart failure (I50)
Calcium channel blockers	C08C, C08D	
ACE-inhibitors †	C09A, C09B	Heart failure (I50)
Angiotensin receptor blockers ‡	C09C, C09DA, C09DB	Heart failure (I50)
Other drugs targeting blood vessels	C02C, C02D	

Patients with a pick-up of a prescription of the anti-hypertensive drugs within the preceding 12 months of the index date were considered as having hypertension. Beta-blockers, ACE-inhibitors, and Angiotensin receptor blockers may be prescribed for other diagnoses than hypertension. Patients were not classified as having hypertension if these drugs were found in combinations with any such diagnosis. An existing record of hypertension (I109) was superior to the pick-ups of prescribed drugs.

* Patients with a diagnosis of angina pectoris (I208, I209), atrial fibrillation (I48), MI (I21, I22) or heart failure (I50) and simultaneously prescribed with beta-blockers were not classified as having hypertension.

† ‡ Patients with a diagnosis of heart failure (I50) with concurrently prescription of ACE inhibitors or angiotensin receptor blockers were not classified as having hypertension.

Svensson P, *et al. BMJ Open* 2022; 11:e044486. doi: 10.1136/bmjopen-2020-044486

eTable 2 - Missing Data[*] in the Study Cohort

Characteristic	Missing data, No. (%)
Age	0 (0.0)
Sex	0 (0.0)
Level of education	239 (2.0)
Region of birth	5 (0.1)
Fills of prescriptions	0 (0)
Medical history	0 (0)

Svensson P, *et al. BMJ Open* 2022; 11:e044486. doi: 10.1136/bmjopen-2020-044486

eTable 3a. Baseline characteristics of the study population in the youngest tertile. Characteristics of patients with Covid-19 requiring mechanical ventilation and control subjects

Youngest tertile (age 21-56 years)

	**Covid-19 (n = 367)**	**Control subjects (n = 3,670)**
Age, median (IQR), y	49 (42-53)	49 (42-53)
**Sex:**		
Male, No. (%)	261 (71.1)	2,610 (71.1)
**Sociodemographics** No. (%)		
Education (years)		
≤9	78 (22.5)	529 (14.8)
10-12	175 (50.4)	1,633 (45.7)
≥12	94 (27.1)	1,409 (39.5)
Marital status		
Unmarried	169 (46.0)	1,911 (52.1)
Married	198 (54.0)	1,759 (47.9)
Region of birth		
EU 15* and/or Nordics	173 (47.3)	2,498 (68.1)
**Medical history** No. (%)		
Type 1 diabetes	3 (0.8)	22 (0.6)
Type 2 diabetes	78 (21.3)	161 (4.4)
Obesity	64 (17.4)	105 (2.9)
Hypertension	102 (27.8)	473 (12.9)
Hyperlipidaemia	53 (14.4)	230 (6.3)
Chronic kidney disease	13 (3.5)	14 (0.4)
Cardiovascular disease	9 (2.5)	53 (1.4)
Myocardial infarction	7 (1.9)	35 (1.0)
Ischemic stroke	2 (0.5)	15 (0.4)
Peripheral artery disease	1 (0.3)	13 (0.4)
Heart failure	9 (2.5)	17 (0.5)
Atrial fibrillation	8 (2.2)	34 (0.9)
Deep vein thrombosis	12 (3.3)	31 (0.8)
Pulmonary embolism	2 (0.5)	9 (0.2)
Chronic obstructive pulmonary disease	2 (0.5)	14 (0.4)
Asthma	41 (11.2)	100 (2.7)
Malignancy	21 (5.7)	240 (6.5)
Rheumatoid arthritis	4 (1.1)	22 (0.6)
Systemic inflammatory disease	8 (2.2)	14 (0.4)
Inflammatory bowel disease	3 (0.8)	55 (1.5)

* EU 15 comprises of Austria, Belgium, Denmark, Finland, France, Germany, Greece, Ireland, Italy, Luxembourg, Netherlands, Portugal, Spain, Sweden, United Kingdom. The Nordic countries include Denmark, Finland, Iceland, Norway, and Sweden.

Svensson P, *et al. BMJ Open* 2022; 11:e044486. doi: 10.1136/bmjopen-2020-044486

eTable 3b. Baseline characteristics of the study population in the mid age-group tertile. Characteristics of patients with Covid-19 requiring mechanical ventilation and control subjects

Mid tertile (age 57-67 years)

	**Covid-19 (n = 379)**	**Control subjects (n = 3,790)**
Age, median (IQR), y	62 (59-65)	62 (59-65)
**Sex:**		
Male, No. (%)	282 (74.4)	2,820 (74.4)
**Sociodemographics** No. (%)		
Education (years)		
≤9	94 (25.1)	697 (18.6)
10-12	163 (43.5)	1,745 (46.5)
≥12	118 (31.5)	1,310 (34.9)
Marital status		
Unmarried	151 (39.8)	1,856 (49.0)
Married	228 (60.2)	1,934 (51.0)
Region of birth		
EU 15* and/or Nordics	195 (51.6)	2,914 (76.9)
**Medical history** No. (%)		
Type 1 diabetes	5 (1.3)	10 (0.3)
Type 2 diabetes	113 (29.8)	463 (12.2)
Obesity	12 (3.2)	75 (2.0)
Hypertension	207 (54.6)	1,578 (41.6)
Hyperlipidaemia	114 (30.1)	770 (20.3)
Chronic kidney disease	14 (3.7)	46 (1.2)
Cardiovascular disease	44 (11.6)	311 (8.2)
Myocardial infarction	26 (6.9)	185 (4.9)
Ischemic stroke	11 (2.9)	81 (2.1)
Peripheral artery disease	8 (2.1)	78 (2.1)
Heart failure	15 (4.0)	96 (2.5)
Atrial fibrillation	19 (5.0)	140 (3.7)
Deep vein thrombosis	13 (3.4)	83 (2.2)
Pulmonary embolism	5 (1.3)	40 (1.1)
Chronic obstructive pulmonary disease	12 (3.2)	75 (2.0)
Asthma	42 (11.1)	138 (3.6)
Malignancy	53 (14.0)	538 (14.2)
Rheumatoid arthritis	9 (2.4)	30 (0.8)
Systemic inflammatory disease	11 (2.9)	39 (1.0)
Inflammatory bowel disease	5 (1.3)	52 (1.4)

* EU 15 comprises of Austria, Belgium, Denmark, Finland, France, Germany, Greece, Ireland, Italy, Luxembourg, Netherlands, Portugal, Spain, Sweden, United Kingdom. The Nordic countries include Denmark, Finland, Iceland, Norway, and Sweden.

Svensson P, *et al. BMJ Open* 2022; 11:e044486. doi: 10.1136/bmjopen-2020-044486

eTable 3c. Baseline characteristics of the study population in the oldest tertile. Characteristics of patients with Covid-19 requiring mechanical ventilation and control subjects

Oldest tertile (age 68-87 years)

	**Covid-19 (n = 340)**	**Control subjects (n = 3,400)**
Age, median (IQR), y	73 (70-76)	73 (70-76)
**Sex:**		
Male, No. (%)	270 (79.4)	2,700 (79.4)
**Sociodemographics** No. (%)		
Education (years)		
≤9	108 (33.3)	918 (27.5)
10-12	128 (39.5)	1,427 (42.8)
≥12	88 (27.2)	993 (29.7)
Marital status		
Unmarried	134 (39.4)	1,452 (42.7)
Married	206 (60.6)	1,948 (57.3)
Region of birth		
EU 15* and/or Nordics	228 (67.5)	2,999 (88.2)
**Medical history** No. (%)		
Type 1 diabetes	1 (0.3)	7 (0.2)
Type 2 diabetes	96 (28.2)	631 (18.6)
Obesity	20 (5.9)	97 (2.9)
Hypertension	243 (71.5)	2,207 (64.9)
Hyperlipidaemia	124 (36.5)	1,101 (32.4)
Chronic kidney disease	14 (4.1)	86 (2.5)
Cardiovascular disease	60 (17.6)	630 (18.5)
Myocardial infarction	22 (6.5)	341 (10.0)
Ischemic stroke	20 (5.9)	179 (5.3)
Peripheral artery disease	16 (4.7)	159 (4.7)
Heart failure	20 (5.9)	216 (6.4)
Atrial fibrillation	57 (16.8)	415 (12.2)
Deep vein thrombosis	15 (4.4)	94 (2.8)
Pulmonary embolism	6 (1.8)	54 (1.6)
Chronic obstructive pulmonary disease	22 (6.5)	149 (4.4)
Asthma	33 (9.7)	139 (4.1)
Malignancy	15 (4.4)	94 (2.8)
Rheumatoid arthritis	4 (1.2)	44 (1.3)
Systemic inflammatory disease	15 (4.4)	76 (2.2)
Inflammatory bowel disease	10 (2.9)	52 (1.5)

* EU 15 comprises of Austria, Belgium, Denmark, Finland, France, Germany, Greece, Ireland, Italy, Luxembourg, Netherlands, Portugal, Spain, Sweden, United Kingdom. The Nordic countries include Denmark, Finland, Iceland, Norway, and Sweden.

Svensson P, *et al. BMJ Open* 2022; 11:e044486. doi: 10.1136/bmjopen-2020-044486

eTable 4. Baseline characteristics of the study population by sex. Characteristics of patients with Covid-19 requiring mechanical ventilation and control subjects

	**Males**		**Females**	
	**Covid-19 (n = 813)**	**Control subjects (n = 8,130)**	**Covid-19 (n = 273)**	**Control subjects (n = 2,730)**
Age, median (IQR), y	62 (54-70)	62 (54-70)	60 (50-68)	60 (50-68)
**Sociodemographics** No. (%)				
Education (years)				
≤9	204 (25.9)	1,678 (21.0)	76 (29.5)	466 (17.3)
10-12	351 (44.5)	3,606 (45.2)	115 (44.6)	1,199 (44.6)
≥12	233 (29.6)	2,691 (33.7)	67 (26.0)	1,021 (38.0)
Marital status				
Unmarried	314 (38.6)	3,760 (46.2)	140 (51.3)	1,459 (53.4)
Married	499 (61.4)	4,370 (53.8)	133 (48.7)	1,271 (46.6)
Region of birth				
EU 15* and/or Nordics	439 (54.3)	6,250 (76.9)	157 (57.5)	2,161 (79.2)
**Medical history** No. (%)				
Diabetes mellitus	216 (26.6)	1,099 (13.5)	69 (25.3)	195 (7.1)
Obesity	63 (7.7)	218 (2.7)	36 (13.2)	110 (4.0)
Hypertension	419 (51.5)	3,332 (41.0)	128 (46.9)	926 (33.9)
Hyperlipidaemia	103 (12.7)	667 (8.2)	26 (9.5)	131 (4.8)
Chronic kidney disease	26 (3.2)	121 (1.5)	14 (5.1)	25 (0.9)
Cardiovascular disease	91 (11.2)	877 (10.8)	14 (5.1)	115 (4.2)
Myocardial infarction	51 (6.3)	521 (6.4)	4 (1.5)	38 (1.4)
Ischemic stroke	22 (2.7)	225 (2.8)	7 (2.6)	49 (1.8)
Peripheral artery disease	19 (2.3)	216 (2.7)	5 (1.8)	33 (1.2)
Heart failure	30 (3.7)	286 (3.5)	10 (3.7)	43 (1.6)
Atrial fibrillation	53 (6.5)	510 (6.3)	12 (4.4)	79 (2.9)
Deep vein thrombosis	31 (3.8)	163 (2.0)	9 (3.3)	45 (1.6)
Pulmonary embolism	9 (1.1)	81 (1.0)	4 (1.5)	22 (0.8)
Chronic obstructive pulmonary disease	24 (3.0)	184 (2.3)	8 (2.9)	53 (1.9)
Asthma	58 (7.1)	256 (3.1)	42 (15.4)	120 (4.4)
Malignancy	122 (15.0)	1,287 (15.8)	36 (13.2)	453 (16.6)
Rheumatoid arthritis	9 (1.1)	54 (0.7)	8 (2.9)	42 (1.5)
Systemic inflammatory disease	13 (1.6)	64 (0.8)	20 (7.3)	65 (2.4)
Inflammatory bowel disease	12 (1.5)	109 (1.3)	5 (1.8)	50 (1.8)

* EU 15 comprises of Austria, Belgium, Denmark, Finland, France, Germany, Greece, Ireland, Italy, Luxembourg, Netherlands, Portugal, Spain, Sweden, United Kingdom. The Nordic countries include Denmark, Finland, Iceland, Norway, and Sweden.

eTable 5. Baseline characteristics of the study population by region of birth. Characteristics of patients with Covid-19 requiring mechanical ventilation and control subjects

	**Region of birth within EU15/ and/or Nordic Countries**		**Region of birth outside EU15/ and/or Nordic Countries**	
	**Covid-19 (n = 596)**	**Control subjects (n = 5,960)**	**Covid-19 (n = 490)**	**Control subjects (n = 4,900)**
Age, median (IQR), y	64 (54-72)	64 (54-72)	59 (51-67)	59 (51-67)
**Sex:**				
Male, No. (%)	439 (73.7)	4,390 (73.7)	374 (76.3)	3,740 (76.3)
**Sociodemographics** No. (%)				
Education (years)				
≤9	121 (20.6)	1,161 (19.8)	159 (34.6)	983 (20.5)
10-12	295 (50.3)	2,636 (44.9)	171 (37.3)	2,169 (45.2)
≥12	171 (29.1)	2,069 (35.3)	129 (28.1)	1,643 (34.3)
Marital status				
Unmarried	299 (50.2)	2,859 (48.0)	155 (31.6)	2,360 (48.2)
Married	297 (49.8)	3,101 (52.0)	335 (68.4)	2,540 (51.8)
**Medical history** No. (%)				
Diabetes mellitus	128 (21.5)	699 (11.7)	157 (32.0)	595 (12.1)
Obesity	60 (10.1)	170 (2.9)	39 (8.0)	158 (3.2)
Hypertension	313 (52.5)	2,547 (42.7)	234 (47.8)	1,711 (34.9)
Hyperlipidaemia	75 (12.6)	496 (8.3)	54 (11.0)	302 (6.2)
Chronic kidney disease	21 (3.5)	83 (1.4)	19 (3.9)	63 (1.3)
Cardiovascular disease	57 (9.6)	622 (10.4)	48 (9.8)	370 (7.6)
Myocardial infarction	29 (4.9)	338 (5.7)	26 (5.3)	221 (4.5)
Ischemic stroke	16 (2.7)	174 (2.9)	13 (2.7)	100 (2.0)
Peripheral artery disease	15 (2.5)	158 (2.7)	9 (1.8)	91 (1.9)
Heart failure	21 (3.5)	192 (3.2)	19 (3.9)	137 (2.8)
Atrial fibrillation	53 (8.9)	373 (6.3)	12 (2.4)	216 (4.4)
Deep vein thrombosis	25 (4.2)	112 (1.9)	15 (3.1)	96 (2.0)
Pulmonary embolism	8 (1.3)	64 (1.1)	5 (1.0)	39 (0.8)
Chronic obstructive pulmonary disease	18 (3.0)	125 (2.1)	14 (2.9)	112 (2.3)
Asthma	53 (8.9)	212 (3.6)	47 (9.6)	164 (3.3)
Malignancy	105 (17.6)	1,075 (18.0)	53 (10.8)	665 (13.6)
Rheumatoid arthritis	9 (1.5)	64 (1.1)	8 (1.6)	32 (0.7)
Systemic inflammatory disease	21 (3.5)	77 (1.3)	12 (2.4)	52 (1.1)
Inflammatory bowel disease	14 (2.3)	89 (1.5)	3 (0.6)	70 (1.4)

* EU 15 comprises of Austria, Belgium, Denmark, Finland, France, Germany, Greece, Ireland, Italy, Luxembourg, Netherlands, Portugal, Spain, Sweden, United Kingdom. The Nordic countries include Denmark, Finland, Iceland, Norway, and Sweden.

eTable 6. Odds ratios for Covid-19 requiring ICU-admission by cardiometabolic factors and other comorbidities (n=1,417 cases and n=14,170 control subjects).

	Adjusted for age and sex			Adjusted model 2†			Adjusted model 3‡		
Risk factors	OR	95% CI	p-value	OR	95% CI	p-value	OR	95% CI	p-value
Type 1 diabetes	2.18	1.19-3.98	0.011	2.84	1.55-5.21	0.001	2.83	1.51-5.30	0.001
Type 2 diabetes	2.88	2.51-3.31	<0.001	2.42	2.09-2.79	<0.001	1.94	1.64-2.31	<0.001
Obesity	4.17	3.46-5.02	<0.001	4.12	3.39-5.02	<0.001	2.91	2.34-3.60	<0.001
Hypertension	1.83	1.61-2.09	<0.001	1.82	1.59-2.08	<0.001	1.41	1.21-1.65	<0.001
Hyperlipidaemia	1.56	1.36-1.79	<0.001	1.47	1.28-1.70	<0.001	0.87	0.74-1.03	0.104
CKD	3.22	2.39-4.33	<0.001	2.91	2.12-3.99	<0.001	1.98	1.36-2.86	<0.001
CVD	1.18	0.98-1.42	0.089	1.12	0.93-1.36	0.240	0.72	0.58-0.90	0.004
Heart failure	1.62	1.24-2.12	<0.001	1.53	1.15-2.03	0.003	0.87	0.62-1.24	0.442
Atrial fibrillation	1.53	1.24-1.90	<0.001	1.69	1.36-2.10	0.001	1.32	1.02-1.71	0.035
VTE	1.49	1.12-1.99	0.006	1.62	1.21-2.17	<0.001	1.39	1.02-1.90	0.039
COPD	1.89	1.42-2.52	<0.001	1.85	1.37-2.49	<0.001	1.07	0.74-1.54-	0.720
Asthma	3.33	2.76-4.02	<0.001	3.39	2.79-4.13	<0.001	2.91	2.35-3.62	<0.001
Malignancy	0.99	0.85-1.16	0.938	1.08	0.92-1.26	0.358	0.98	0.83-1.16	0.837
Rheumatoid arthritis	2.08	1.35-3.18	0.001	2.22	1.45-3.39	<0.001	1.68	1.03-2.74	0.038
Systemic infl. disease	2.60	1.85-3.64	<0.001	2.51	1.77-3.57	<0.001	1.85	1.27-2.70	0.001
Infl. bowel disease	0.98	0.62-1.53	0.917	1.10	0.69-1.75	0.688	1.00	0.60-1.64	0.986

†Adjusted for age, sex, educational level, marital status and region of birth.
‡Adjusted for age, sex, educational level, marital status and region of birth and all diagnoses in table 2a.

Supplemental Fig. S1 – Directed Acyclic Graph

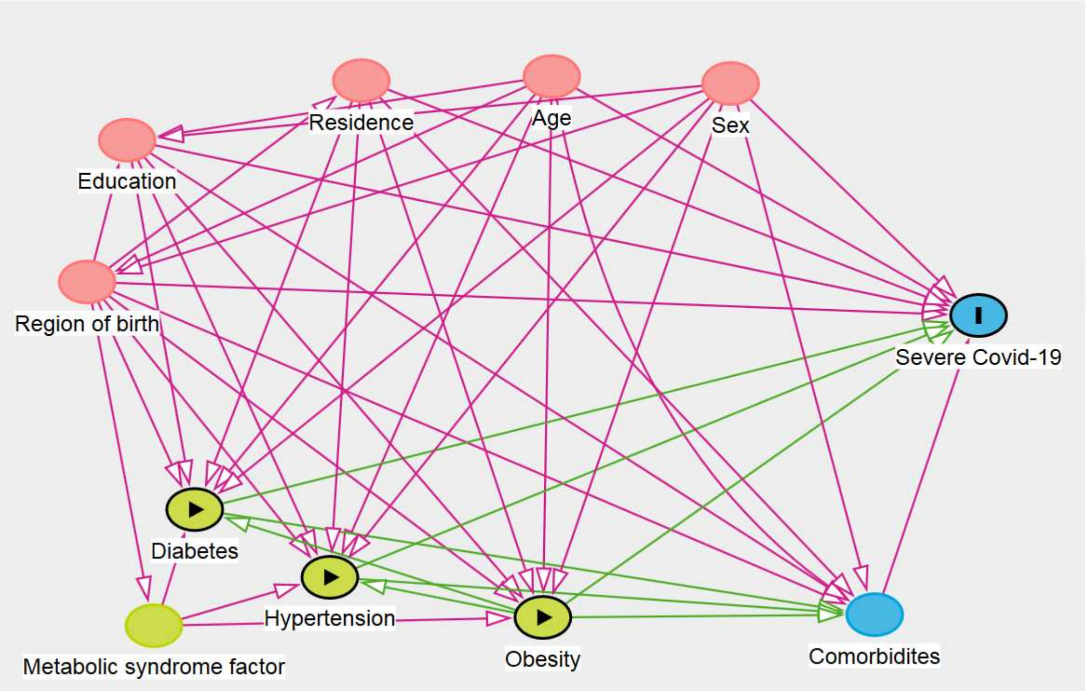

Supplemental Fig. S2 - Flow chart of exclusion criteria

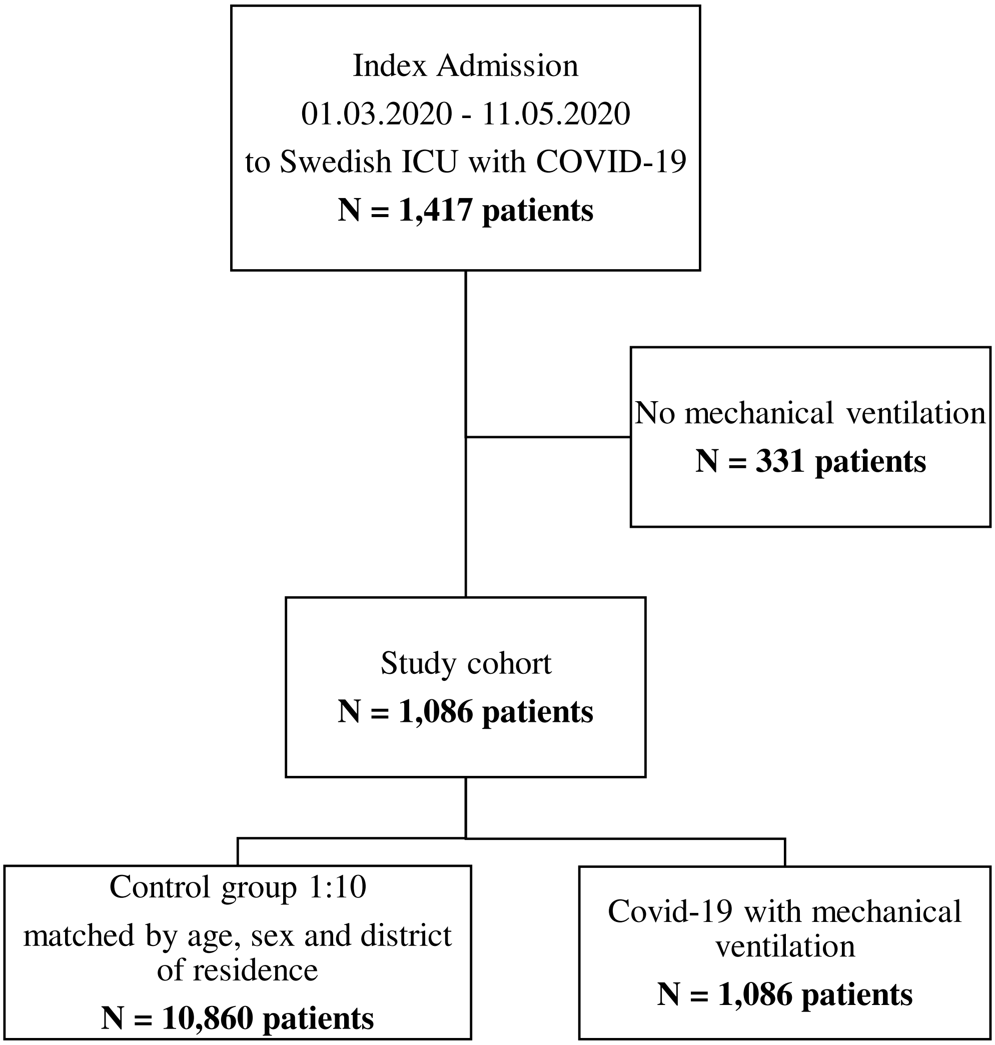

1. Ludvigsson JF, Andersson E, Ekbom A, Feychting M, Kim JL, Reuterwall C, Heurgren M and Olausson PO. External review and validation of the Swedish national inpatient register. *BMC Public Health*. 2011;11:450.

Svensson P, *et al. BMJ Open* 2022; 11:e044486. doi: 10.1136/bmjopen-2020-044486
