# Supplementary material for: Association between cardiometabolic disease and severe COVID-19: a nationwide case–control study of patients requiring invasive mechanical ventilation
Source: BMJ Open. 2021 Feb 17;11(2):e044486. doi: 10.1136/bmjopen-2020-044486 (PMC7893210; doi:10.1136/bmjopen-2020-044486)
Supplement: Author's manuscript [file bmjopen-2020-044486.draft_revisions.pdf]

BMJ Open is committed to open peer review. As part of this commitment we make the peer review history of every article we publish publicly available.

When an article is published we post the peer reviewers' comments and the authors' responses online. We also post the versions of the paper that were used during peer review. These are the versions that the peer review comments apply to.

The versions of the paper that follow are the versions that were submitted during the peer review process. They are not the versions of record or the final published versions. They should not be cited or distributed as the published version of this manuscript.

BMJ Open is an open access journal and the full, final, typeset and author-corrected version of record of the manuscript is available on our site with no access controls, subscription charges or pay-per-view fees (http://bmjopen.bmj.com).

If you have any questions on BMJ Open's open peer review process please email info.bmjopen@bmj.com

BMJ Open

Cardiometabolic factors and risk for severe Covid-19 requiring invasive mechanical ventilation during the Swedish epidemic

Journal:	*BMJ Open*
Manuscript ID	bmjopen-2020-044486
Article Type:	Original research
Date Submitted by the Author:	05-Sep-2020
Complete List of Authors:	Svensson, Per; Karolinska Institutet Department of Clinical Science and Education Sodersjukhuset,<br>Hofmann, Robin; Karolinska Institute, Department of Clinical Science and Education, Södersjukhuset<br>Häbel, Henrike; Institute of Environmental Medicine<br>Jernberg, Tomas; Danderyd University Hospital, Karolinska Institutet<br>Nordberg, Per; Karolinska institutet, Dept. of Cardiology, Centre for Reuscitation Science
Keywords:	COVID-19, Diabetes & endocrinology < INTERNAL MEDICINE, Hypertension < CARDIOLOGY, INTENSIVE & CRITICAL CARE

SCHOLARONE™
Manuscripts

I, the Submitting Author has the right to grant and does grant on behalf of all authors of the Work (as defined in the below author licence), an exclusive licence and/or a non-exclusive licence for contributions from authors who are: i) UK Crown employees; ii) where BMJ has agreed a CC-BY licence shall apply, and/or iii) in accordance with the terms applicable for US Federal Government officers or employees acting as part of their official duties; on a worldwide, perpetual, irrevocable, royalty-free basis to BMJ Publishing Group Ltd ("BMJ") its licensees and where the relevant Journal is co-owned by BMJ to the co-owners of the Journal, to publish the Work in this journal and any other BMJ products and to exploit all rights, as set out in our licence.

The Submitting Author accepts and understands that any supply made under these terms is made by BMJ to the Submitting Author unless you are acting as an employee on behalf of your employer or a postgraduate student of an affiliated institution which is paying any applicable article publishing charge ("APC") for Open Access articles. Where the Submitting Author wishes to make the Work available on an Open Access basis (and intends to pay the relevant APC), the terms of reuse of such Open Access shall be governed by a Creative Commons licence – details of these licences and which Creative Commons licence will apply to this Work are set out in our licence referred to above.

Other than as permitted in any relevant BMJ Author's Self Archiving Policies, I confirm this Work has not been accepted for publication elsewhere, is not being considered for publication elsewhere and does not duplicate material already published. I confirm all authors consent to publication of this Work and authorise the granting of this licence.

For peer review only

Cardiometabolic factors and risk for severe Covid-19 requiring invasive mechanical ventilation during the Swedish epidemic

Per Svensson, MD, PhD[1,2]; Robin Hofmann, MD, PhD[1,2]; Henrike Häbel, PhD[3]; Tomas Jernberg, MD, PhD [4]; and Per Nordberg, MD, PhD[5,2]

1. Department of Clinical Science and Education, Södersjukhuset, Karolinska Institutet, Stockholm, Sweden
2. Department of Cardiology, Södersjukhuset, Stockholm, Sweden
3. Institute of Environmental Medicine, Division of Biostatistics, Karolinska Institutet, Stockholm, Sweden
4. Department of Clinical Sciences, Danderyd University Hospital, Karolinska Institutet, Stockholm, Sweden
5. Department of Medicine Solna, Karolinska Institutet, Stockholm, Sweden

Word count: 4637

Correspondence to: Per Svensson
Assoc. Prof, MD, FESC
Department of Clinical Science and Education, Södersjukhuset, Karolinska Institutet
Department of Cardiology, Södersjukhuset
Sjukhusbacken 10, SE 118 83 Stockholm, Sweden. +46 (0)8 616 3014
Email: per.svensson@ki.se
Twitter: @perpersvensson

For peer review only - http://bmjopen.bmj.com/site/about/guidelines.xhtml

ABSTRACT

Aims:

The risks associated with diabetes, obesity and hypertension for severe Covid-19 may be confounded and differ by sociodemographic background. We assessed the risks associated with cardiometabolic factors for severe Covid-19 when accounting for socioeconomic factors and in subgroups by age, sex and region of birth.

Methods and results

In this nationwide case-control study, 1.086 patients admitted to intensive care with Covid-19 requiring mechanical ventilation (cases) and 10.860 population-based controls matched for age, sex and district of residency were included from mandatory national registries. Odds Ratios (ORs) with 95% confidence intervals (CIs) for associations between severe Covid-19 and exposures with adjustment for confounders were estimated using logistic regression. The median age was 62 years (IQR 52-70), and 3,003 (24.9%) were female. Type 2 diabetes (OR, 2.3 [95% CI, 1.9-2.7]), hypertension (OR, 1.7 [95% CI, 1.5-2.0]), obesity (OR, 3.1 [95% CI, 2.4-4.0]) and chronic kidney disease (OR, 2.5 [95% CI, 1.7-3.7]) were all associated with severe Covid-19. In the younger subgroup (below 62 years) ORs were significantly higher for all cardiometabolic risk factors. The risk associated with type 2 diabetes was higher in women (p=0.001) and in patients with a region of birth outside EU (p=0.004).

Conclusion

Diabetes, obesity and hypertension were all independently associated with severe Covid-19 with stronger associations in the younger population. Type 2 diabetes implied a greater risk among women and in non-EU immigrants. These findings, originating from high-quality

For peer review only

Swedish registries, may be important to direct preventive measures such as vaccination to susceptible patient groups.

Trial registration: Clinicaltrial.gov (NCT04426084)

For peer review only

For peer review only - http://bmjopen.bmj.com/site/about/guidelines.xhtml

STRENGTH AND LIMITATIONS OF THIS STUDY

- In difference to many previous reports, this study accounts for severity of Covid-19 and provide a homogeneous study group, by only including intubated patients at the intensive care with the highest risk of death. By inclusion of virtually all such cases nationwide, in a country with a tax-financed health care and a serious epidemic during the study period, the study provides a large sample size with adequate power and high external validity.
- This study compared severe Covid-19 patients with matched controls in the underlying population and used 10 population-based, age, sex and district of residence matched controls per each case. Therefore the study can provide estimates of relative risk for severe Covid-19 in the population for the studied risk factors.
- Socioeconomic factors have been crucial in how the pandemic has impacted on different groups in the society but are also closely linked with obesity, diabetes and cardiovascular disease. By matching for district of residence and adjusting for individual level data on several socioeconomic variables this study provide novel evidence that diabetes, obesity and hypertension are independently associated with severe Covid-19, but also that the relative importance of risk factors differ by age, sex and region of birth.
- The data on exposures are from high quality national registries with national coverage and the patient cohort is the most complete Swedish cohort on severe Covid-19 published to date. The findings may be important to direct preventive measures such as vaccination to susceptible patient groups.

For peer review only

INTRODUCTION

Observational data suggest both a higher prevalence, and a more severe course of corona virus disease 2019 (Covid-19) among individuals with diabetes, obesity and hypertension,[1-5] risk factors that are closely linked and cluster together in the metabolic syndrome.[6] As more clinical data are emerging, new determinants of both Covid-19 and severity of disease are being discussed, such as coagulation disorders[7] and socio-economic factors[8] but to this point the underlying mechanisms that link cardiometabolic disease with severe Covid-19 are unclear.[9]

In order to advance the knowledge on risk factors, several aspects are crucial to put evidence into perspective: First, the spectrum of disease severity needs to be addressed as the clinical presentation of infected patients can range from asymptomatic, to severe with high risk of fatal outcome. As the risks of being infected may differ from the risk of becoming severely ill once infected, there is a need for studies that focus on risk factors associated with a severe disease. Second, as socio-economic and cultural factors are closely linked to type 2 diabetes, obesity and cardiovascular disease, these need to be accounted for in such analyses. And, most importantly, prevalent cases need to be compared to controls to reliably assess the magnitude of the major risk factors in the underlying population.[10] To our knowledge, no study has investigated whether cardiometabolic risk factors are independently associated with severe Covid-19, when controlled for age, sex, sociodemographic factors, and immigrant background using matched population-based controls. In addition, it is unknown whether the impact of these cardiometabolic risk factors is attenuated by age, sex and sociodemographic factors.

Sweden has been hit hard by the Covid-19 epidemic but in contrast to most other countries, did not employ a strict lock-down policy. To continuously evaluate the situation, strong governmental efforts were enforced on national health care registries for data reporting.

For peer review only

In this study we present a comprehensive Swedish sample originating from mandatory, high-quality national registries with the aim to investigate whether cardiometabolic risk factors are associated with severe Covid-19 in patients treated at the intensive care unit with invasive mechanical ventilation.

METHODS

Study design and ethics

This nationwide case-control study was based on data from the Swedish Intensive Care Registry (SIR) on patients (cases) with severe Covid-19 admitted to the ICU requiring invasive mechanical ventilation between 1st of March until 11th of May 2020. For each case, 10 controls were randomly selected from the Swedish Population Register and matched by age, sex and district of residence (corresponding to part of municipality). The study database was merged with multiple mandatory Swedish national registries at Statistics Sweden and the National Board of Health and Welfare using each individual's unique personal identification number.[11] The study complied with the Declaration of Helsinki, was approved by the National Ethical Review Board (identification number 2020/124-31/4) and registered at Clinicaltrial.gov (NCT04426084). The study used already collected, pseudonymized data and involved minimal infringement of personal integrity.

Patient and Public Involvement

Patients and the public were not involved in the design, or conduct of our research.

National registries and Data collection

For peer review only - http://bmjopen.bmj.com/site/about/guidelines.xhtml
For peer review only

Severe COVID-19 was defined as laboratory confirmed COVID-19 infection in individuals treated at the intensive care unit (ICU) with mechanical ventilation. These cases were reported to SIR[12] which is a national register with about 95% coverage of all ICU admissions in Sweden and was used to identify eligible patients. The National Patient Register[13] was used to collect primary or secondary diagnoses from previous hospital admissions and outpatients' visits coded according to the International Classification of Diseases (ICD) version 10 within the 15 years preceding the admission. The Prescribed Drugs Register contains information on all dispensed drugs according to the Anatomical Therapeutic Chemical Classification (ATC). We collected individual data on dispensed drugs prescribed and claimed within 12 months before the study period. The longitudinal integrated database for health insurance and labor market studies is managed by Statistics Sweden and includes annual measurements on several socioeconomic and sociodemographic variables, including income, education and country of birth.[14]

Definition of Exposures and Outcomes

Exposures were a history of cardiometabolic or relevant chronic disease including hypertension (defined as previous diagnosis of ICD I10 or prescription of antihypertensive drugs within the preceding 12 months as described previously[15]), hyperlipidemia (ICD E78 or prescription of lipid lowering drugs within the preceding 12 months), diabetes mellitus type 2(ICD E11 or prescription of antidiabetic drugs within the preceding 12 months), diabetes mellitus type 1 (ICD E10), obesity (ICD E66), heart failure (ICD I50.1, I50.9), atrial fibrillation (ICD I48), venous thromboembolism (ICD I26, I80), asthma (ICD J45), chronic obstructive pulmonary disease (ICD J44), chronic kidney disease (ICD N18), malignancy (ICD C, D40-48), rheumatoid arthritis (ICD M05, M06), systemic inflammatory disease (ICD M30-M36), and inflammatory bowel disease (ICD K50, K51). A history of cardiovascular

For peer review only - http://bmjopen.bmj.com/site/about/guidelines.xhtml

disease (CVD) was defined as a record of either MI (ICD I21, I22), ischemic heart disease (ICD I25), ischemic stroke (ICD I63), or peripheral vascular disease (ICD I70-I73), in the Swedish Patient Register (Supplementary eTable1).

Definition of covariates and variables for subgroup analyses

Level of education was categorized as ≤9 years (reference), 10-12 years, and >12 years based on the highest educational level attained during the year before admission. Region of birth was categorized as a country of birth within EU15 (Austria, Belgium, Denmark, Finland, France, Germany, Greece, Ireland, Italy, Luxembourg, Netherlands, Portugal, Spain, Sweden, United Kingdom) and/or the Nordic Countries (Denmark, Finland, Iceland, Norway, Sweden), or having a country of birth outside this region. Marital status during the year before index date was categorized as married or not married which included unmarried, divorced, and widowed. Subgroup analyses were performed for region of birth, sex (male/female) and age (above/below median age).

Outcome

The outcome was defined as an ICU admission due to Covid-19, registered in SIR, with at least one episode of invasive mechanical ventilation during the ICU stay. All eligible patients during the study period were included as cases in the study.

Statistical Methods

Categorical variables are reported as frequencies and percentages, while continuous variables are reported as median and interquartile range (IQR). Missing data are reported in Supplemental eTable 2. Odds ratios (OR) and 95% confidence intervals (CI) for the association between the different exposures and the outcome were calculated by means of

For peer review only - http://bmjopen.bmj.com/site/about/guidelines.xhtml
For peer review only

logistic regression adjusted for age and sex (Model 1). For all exposures additional adjustments were made for sociodemographic and socioeconomic variables (marital status, region of birth and educational level) (Model 2) and, finally, for all conditions in table 2a that were used as covariates in a fully adjusted regression model (Model 3) in order to analyse both total effects unconfounded of sociodemographic variables and direct effects in accordance to our perception of causal relationships as illustrated by the directed acyclic graphs in supplementary figure S1. Standard errors were calculated using the robust sandwich estimator and the significance level was set at an alpha of 0.05. For a formal test of a significant difference between the ORs for different subgroups, likelihood-ratio tests were conducted between a model with and without an interaction term between the indicator variable for the subgroup and the risk factor. For these tests, the robust sandwich estimator was not used in the underlying logistic regression models.

Statistical analyses were performed using Stata version 16.0 (StataCorp, College Station, TX).

For peer review only

RESULTS

During the study period between 1st of March until 11th of May 2020, a total of 1,417 patients were admitted to an intensive care unit in Sweden due to Covid-19 out of which 1,086 required treatment with invasive mechanical ventilation (cases). For each case, 10 matched control subjects were randomly selected, rendering a total of 10,860 control subjects. The study population selection procedure and reasons for exclusions are described in Supplemental Figure S2.

Patient characteristics

The median age was 62 (IQR 52-70) years and 75% were men. Baseline characteristics are summarized in Table 1a. Patients were less likely to have a post-secondary education and more likely to be married compared to the control group. Further, patients were more likely to have history of migration with more patients having a region of birth outside EU15 and the Nordic countries. Comorbid conditions were more common among patients with Covid-19 receiving mechanical ventilation compared to the control group. In particular, cardiometabolic risk factors were overrepresented with more patients having a history of hypertension, hyperlipidemia, diabetes mellitus, obesity and chronic kidney disease, but also venous thromboembolic disease, asthma and systemic inflammatory diseases were more common. Due to more comorbid conditions, patients had correspondingly more pharmacological treatments (table 1b). All antihypertensive treatments were more common among patients compared to controls subjects as were all antidiabetic treatments except meglitinides.

Comparison of risk factors and treatments between patient with severe Covid-19 and controls

For peer review only - http://bmjopen.bmj.com/site/about/guidelines.xhtml

In the multivariable logistic regression models presented in table 2a, both diabetes, hypertension, hyperlipidemia, obesity and chronic kidney disease were associated with Covid-19 receiving mechanical ventilation. All associations remained significant after adjustment for possible socioeconomic confounders. In the fully adjusted model, all cardiometabolic risk factors except hyperlipidemia were associated with the outcome indicating direct and additive effects for these risk factors (figure 1). In addition, we observed associations between a history of venous thromboembolic disease, asthma, rheumatoid arthritis, as well as systemic inflammatory disease and severe Covid-19. In contrast, neither cardiovascular disease, heart failure, atrial fibrillation, malignancy, chronic obstructive pulmonary disease or inflammatory bowel disease were associated with the outcome.

In the logistic regression analysis adjusted for age and sex using no treatment as reference, all types of antihypertensive treatment, except diuretics and all types of antidiabetic treatments, except meglinitides, were associated with severe Covid-19 (table 2b). However, when adjusting for all comorbidities in the fully adjusted model, calcium-channel blockers, biguanides and glitazones were the only treatments which remained associated with the outcome.

Subgroup analysis

Baseline characteristics by subgroups of age, sex and region of birth are summarized in supplemental eTable 3, eTable 4 and eTable 5 respectively. A regression analysis for the cardiometabolic risk factors is presented in table 3 by subgroups of age, sex and region of birth. In the younger subgroup (defined as age below median of 62), odds ratios were significantly higher for hypertension (p-value for interaction, p<0.001), type 2 diabetes (p<0.001), obesity (p=0.027), cardiovascular disease (p=0.013), chronic kidney disease (p=0.010), and asthma (p=0.022) as illustrated in figure 2. In women, the odds ratios for type

For peer review only - http://bmjopen.bmj.com/site/about/guidelines.xhtml
For peer review only

2 diabetes (p=0.001) and asthma (p=0.030) were significantly higher as compared to men (figure 3). Among patients with a region of birth outside EU 15, diabetes had a stronger association with severe Covid-19 compared to patients with a region of birth within EU15 (p=0.004), whereas a trend towards the opposite was observed for obesity (figure 4).

For peer review only

For peer review only - http://bmjopen.bmj.com/site/about/guidelines.xhtml

DISCUSSION

In the present nationwide case-control study, assessing the risk for severe Covid-19 with need for mechanical ventilation at the intensive care unit, we found that the cardiometabolic risk factors diabetes, obesity and hypertension, were strongly and independently associated with severe infection also when accounting for socioeconomic factors. Furthermore, we found higher risks associated with all cardiometabolic risk factors among younger patients whereas diabetes was more important in women and in those with an immigrant background. These findings, originating from high-quality national registries in Sweden which has been experiencing a serious epidemic, may be important to identify susceptible patient groups requiring extra precautions and prioritized for vaccination.

Cardiometabolic risk factors were early linked with a severe Covid-19 in case-series[16] and uncontrolled studies[17] and later in studies using population-based control subjects.[2] Here, we confirm these findings and extend them to patients with severe disease in a well-controlled design. We can reinforce that diabetes, obesity and hypertension - risk factors that are closely linked and often cluster together in the metabolic syndrome[18] - all have strong and independent direct associations with the outcome. By adjusting for sociodemographic factors, we can also show for the first time that obesity and other components of the metabolic syndrome - factors that are closely linked with lower socioeconomic status[19] - act on Covid-19 independent of sociodemography. The effect of diabetes was even stronger in the population with an immigrant background.

Previous studies on hypertension as a risk factor for severe disease[20] may have been confounded by age and until now, there has been limited evidence of hypertension being an independent risk factor.[21] We are aware of only one major study that used population-based

For peer review only - http://bmjopen.bmj.com/site/about/guidelines.xhtml

controls, but that study did not report associations of hypertension with severe disease.[2] Here, we can report that hypertension is not only a risk factor independent of age and other related conditions, but also that risk factor patterns differ by age, sex, and region of birth. In the younger subgroup (age below 62 years) all cardiometabolic factors had an even stronger association with severe Covid-19. In women and individuals born outside EU 15, diabetes had the strongest association with the outcome. All components of metabolic syndrome are associated with endothelial dysfunction[22] and low-grade inflammation.[23] Hypertension is also linked with a dysregulated immune system,[24] including endothelial mechanisms,[25] and is causally associated with increased lymphocyte count,.[26] As emerging evidence suggests that endothelial inflammation is involved in serious manifestations of Covid-19,[27] it is possible that a common mechanism linking cardiometabolic risk factors with severe Covid-19 is mediated through endothelial and microcirculatory dysfunction. Our findings suggest that these mechanisms are even more important at a younger age.

We identified asthma, previous thromboembolic disease, rheumatoid arthritis, and systemic inflammatory disease as additional chronic diseases with increased risk for a severe course of Covid-19. This is new and important information as patients with chronic inflammatory conditions may also be more susceptible to the proinflammatory pathways of the infection that involves diffuse endothelial inflammation and systemic impaired microcirculation leading to multiorgan dysfunction.[27]

The association between socioeconomic factors and cardiovascular disease is well established[19] and socioeconomic factors have also been important during the Covid-19-pandemic.[28 29] We therefore believe that matching for residency, which is linked with

For peer review only - http://bmjopen.bmj.com/site/about/guidelines.xhtml

socioeconomic factors, as well as adjustments for individual level information on migration, level of education and marital status, is a crucial factor in our study design.

In comparison to several previous descriptive studies we have a well-characterised, homogenous, nationwide population with severe disease, all needing mechanical ventilation at the intensive care unit. To the best of our knowledge, this is the first study that includes virtually all cases with severe disease together with a population based matched control group. In addition, most previous studies include a heterogeneous mix of cases where other factors such as testing patterns in mild cases may have influenced overall results.[2] Consequently, the current study estimated relative risks in the population for developing severe Covid-19 which may differ from the risk of obtaining an infection with a milder course of disease. Since Swedish health care is virtually fully tax funded, all acute treatments including admission to the ICU with invasive mechanical ventilation, are based on medical decisions and do not involve private-economic considerations. Unequal access to health care is thereby reduced, increasing the validity of our results. There are some limitations with the present study. First, in terms of external validity, patient selection for intensive care treatment including invasive ventilation may differ between countries,[30] however, it is unlikely that this will affect the relative importance of risk factors. Second, we did not have any information concerning the rate of mild infection with Covid-19 among control subjects. Finally, the observational design of the study cannot exclude the potential of residual confounding and the results should be interpreted as such.

For peer review only

CONCLUSIONS

Diabetes, obesity and hypertension were all independently associated with severe Covid-19 requiring mechanical ventilation at the intensive care unit, with strongest associations in the younger population. Type 2 diabetes implied a greater risk among women and in those with immigrant background. These findings, originating from high-quality Swedish registries, may be important to direct preventive measures such as vaccination to susceptible patient groups.

For peer review only

For peer review only - http://bmjopen.bmj.com/site/about/guidelines.xhtml

Funding

This work was supported by grants from the Region Stockholm (ALF-project, grant number 2019-0100).

RH was supported by Region Stockholm (clinical postdoctoral appointment, grant number K 2017-4577) and the Swedish Heart Lung foundation (grant number 20180187)

For peer review only

ACKNOWLEDGMENTS

Authors' contributions

PS had full access to all the data in the study and takes full responsibility for the integrity of the data and accuracy of the data analysis.

Study concept and design: PS, PN, HH.

Acquisition of data: PS

Analysis and interpretation of data: All authors

Drafting the manuscript: PS, RH, PN.

Critical revision of the manuscript for important intellectual content: All authors

Statistical analysis: HH.

Obtained funding: PS

Disclosures

The authors declare that there is no conflict of interest.

Data availability

For peer review only - http://bmjopen.bmj.com/site/about/guidelines.xhtml

The data underlying this article cannot be shared publicly due to the privacy of individuals that participated in the study. The data will be shared on reasonable request to the corresponding author.

For peer review only

For peer review only - http://bmjopen.bmj.com/site/about/guidelines.xhtml

REFERENCES

1. Zhou F, Yu T, Du R, et al. Clinical course and risk factors for mortality of adult inpatients with COVID-19 in Wuhan, China: a retrospective cohort study. *Lancet (London, England).* 2020.
2. Mancia G, Rea F, Ludergnani M, Apolone G, Corrao G. Renin–Angiotensin–Aldosterone System Blockers and the Risk of Covid-19. *New England Journal of Medicine.* 2020.
3. Fosbol EL, Butt JH, Ostergaard L, et al. Association of Angiotensin-Converting Enzyme Inhibitor or Angiotensin Receptor Blocker Use With COVID-19 Diagnosis and Mortality. *JAMA.* 2020.
4. Petrilli CM, Jones SA, Yang J, et al. Factors associated with hospital admission and critical illness among 5279 people with coronavirus disease 2019 in New York City: prospective cohort study. *BMJ.* 2020;369:m1966.
5. Sattar N, McInnes IB, McMurray JJV. Obesity a Risk Factor for Severe COVID-19 Infection: Multiple Potential Mechanisms. *Circulation.* 2020.
6. Stefan N. Causes, consequences, and treatment of metabolically unhealthy fat distribution. *The Lancet Diabetes & Endocrinology.* 2020;8(7):616-627.
7. Becker RC. COVID-19 update: Covid-19-associated coagulopathy. *J Thromb Thrombolysis.* 2020;50(1):54-67.
8. Pareek M, Bangash MN, Pareek N, et al. Ethnicity and COVID-19: an urgent public health research priority. *Lancet.* 2020;395(10234):1421-1422.
9. Bornstein SR, Rubino F, Khunti K, et al. Practical recommendations for the management of diabetes in patients with COVID-19. *The Lancet Diabetes & Endocrinology.* 2020;8(6):546-550.

For peer review only - http://bmjopen.bmj.com/site/about/guidelines.xhtml

10. Lipsitch M, Swerdlow DL, Finelli L. Defining the Epidemiology of Covid-19 - Studies Needed. *The New England journal of medicine.* 2020;382(13):1194-1196.

11. Ludvigsson JF, Otterblad-Olausson P, Pettersson BU, Ekbom A. The Swedish personal identity number: possibilities and pitfalls in healthcare and medical research. *European Journal of Epidemiology.* 2009;24(11):659-667.

12. Swedish registry for Intensive Care. National Quality Registry for Intensive Care. http://kvalitetsregister.se/englishpages/findaregistry/registerarkivenglish/nationalqualityregistryforintensivecaresir.2175.html. .

13. Ludvigsson JF, Andersson E, Ekbom A, et al. External review and validation of the Swedish national inpatient register. *BMC Public Health.* 2011;11:450.

14. Ludvigsson JF, Svedberg P, Olén O, Bruze G, Neovius M. The longitudinal integrated database for health insurance and labour market studies (LISA) and its use in medical research. *European Journal of Epidemiology.* 2019;34(4):423-437.

15. Oras P, Habel H, Skoglund PH, Svensson P. Elevated Blood Pressure in the Emergency Department: A Risk Factor for Incident Cardiovascular Disease. *Hypertension (Dallas, Tex : 1979).* 2020;75(1):229-236.

16. Wang D, Hu B, Hu C, et al. Clinical Characteristics of 138 Hospitalized Patients With 2019 Novel Coronavirus–Infected Pneumonia in Wuhan, China. *JAMA.* 2020;323(11):1061-1069.

17. Grasselli G, Zangrillo A, Zanella A, et al. Baseline Characteristics and Outcomes of 1591 Patients Infected With SARS-CoV-2 Admitted to ICUs of the Lombardy Region, Italy. *JAMA.* 2020;323(16):1574-1581.

18. Torpy JM, Lynm C, Glass RM. The Metabolic Syndrome. *JAMA.* 2006;295(7):850-850.

For peer review only

19. Schultz WM, Kelli HM, Lisko JC, et al. Socioeconomic Status and Cardiovascular Outcomes. *Circulation.* 2018;137(20):2166-2178.

20. Gao C, Cai Y, Zhang K, et al. Association of hypertension and antihypertensive treatment with COVID-19 mortality: a retrospective observational study. *European Heart Journal.* 2020;41(22):2058-2066.

21. Kreutz R, Algharably EAE, Azizi M, et al. Hypertension, the renin-angiotensin system, and the risk of lower respiratory tract infections and lung injury: implications for COVID-19. *Cardiovasc Res.* 2020.

22. Tziomalos K, Athyros VG, Karagiannis A, Mikhailidis DP. Endothelial dysfunction in metabolic syndrome: prevalence, pathogenesis and management. *Nutrition, metabolism, and cardiovascular diseases : NMCD.* 2010;20(2):140-146.

23. Hotamisligil GS. Inflammation and metabolic disorders. *Nature.* 2006;444(7121):860-867.

24. Drummond GR, Vinh A, Guzik TJ, Sobey CG. Immune mechanisms of hypertension. *Nature reviews Immunology.* 2019;19(8):517-532.

25. Loperena R, Van Beusecum JP, Itani HA, et al. Hypertension and increased endothelial mechanical stretch promote monocyte differentiation and activation: roles of STAT3, interleukin 6 and hydrogen peroxide. *Cardiovasc Res.* 2018;114(11):1547-1563.

26. Siedlinski M, Jozefczuk E, Xu X, et al. White Blood Cells and Blood Pressure: A Mendelian Randomization Study. *Circulation.* 2020;141(16):1307-1317.

27. Pons S, Fodil S, Azoulay E, Zafrani L. The vascular endothelium: the cornerstone of organ dysfunction in severe SARS-CoV-2 infection. *Critical care (London, England).* 2020;24(1):353.

For peer review only - http://bmjopen.bmj.com/site/about/guidelines.xhtml

28. Price-Haywood EG, Burton J, Fort D, Seoane L. Hospitalization and Mortality among Black Patients and White Patients with Covid-19. *New England Journal of Medicine.* 2020;382(26):2534-2543.

29. Lassale C, Gaye B, Hamer M, Gale CR, Batty GD. Ethnic disparities in hospitalisation for COVID-19 in England: The role of socioeconomic factors, mental health, and inflammatory and pro-inflammatory factors in a community-based cohort study. *Brain, behavior, and immunity.* 2020.

30. Rhodes A, Ferdinande P, Flaatten H, Guidet B, Metnitz PG, Moreno RP. The variability of critical care bed numbers in Europe. *Intensive care medicine.* 2012;38(10):1647-1653.

For peer review only

For peer review only - http://bmjopen.bmj.com/site/about/guidelines.xhtml

FIGURE LEGENDS

Figure 1. Associations of cardiometabolic risk factors with severe Covid-19 (adjusted odds ratios with 95% Cis).

†Adjusted for age, sex, educational level, marital status and region of birth.

‡Adjusted for age, sex, educational level, marital status, region of birth and all diagnoses in table 2a

Figure 2. Associations of cardiometabolic risk factors with severe Covid-19 by age below or over median age of 62 years (adjusted odds ratios with 95% Cis).

P-values denote likelihood-ratio tests between a model with and one without an interaction term between the indicator variable for the subgroup and the risk factor added to model 2

Figure 3. Associations of cardiometabolic risk factors with severe Covid-19 by sex (adjusted odds ratios with 95% Cis).

P-values denote likelihood-ratio tests between a model with and one without an interaction term between the indicator variable for the subgroup and the risk factor added to model 2

Figure 4. Associations of cardiometabolic risk factors with severe Covid-19 by region of birth * (adjusted odds ratios with 95% Cis).

*EU 15 comprises of Austria, Belgium, Denmark, Finland, France, Germany, Greece, Ireland, Italy, Luxembourg, Netherlands, Portugal, Spain, Sweden, United Kingdom. The Nordic countries include Denmark, Finland, Iceland, Norway, and Sweden.

P-values denote likelihood-ratio tests between a model with and one without an interaction term between the indicator variable for the subgroup and the risk factor added to model 2

.

For peer review only

For peer review only - http://bmjopen.bmj.com/site/about/guidelines.xhtml

APPENDICES

Appendix A. Online Supplementary data

Supplemental methods – register information. 1

eTable 1 – Hypertension definitions. 2

eTable 2 - Missing Data in the Study Population. 3

eTable 3 - Baseline characteristics of the study population by age-group. 4

eTable 4 - Baseline characteristics of the study population by sex. 5

eTable 5. Baseline characteristics of the study population by region of birth. 6

Supplemental Figure S1 – Directed Acyclic Graph. 7

Supplemental Figure S2 - Exclusion flowchart. 8

For peer review only

Table 1. Baseline characteristics of the study population. Characteristics of patients with COVID-19 requiring mechanical ventilation and control subjects

	COVID-19 (n = 1,086)	Control subjects (n = 10,860)
Age, median (IQR), y	62.0 (52.0-70.0)	62.0 (52.0-70.0)
**Sex:**		
Male, No. (%)	813 (74.9)	8,130 (74.9)
**Sociodemographics** No. (%)		
Education (years)		
≤9	280 (26.8)	2,144 (20.1)
10-12	466 (44.6)	4,805 (45.1)
≥12	300 (28.7)	3,712 (34.8)
Marital status		
Widow	41 (3.8)	405 (3.7)
Married	632 (58.2)	5,641 (51.9)
Single	218 (20.1)	2,802 (25.8)
Separated	195 (18.0)	2,012 (18.5)
Region of birth		
EU 15* and/or Nordics	596 (55.1)	8,411 (77.5)
**Medical history** No. (%)		
Type 1 diabetes	9 (0.8)	39 (0.4)
Type 2 diabetes	276 (25.4)	1,255 (11.6)
Obesity	99 (9.1)	328 (3.0)
Hypertension	547 (50.4)	4,258 (39.2)
Hyperlipidaemia	292 (33.0)	2,100 (28.6)
Chronic kidney disease	40 (3.7)	146 (1.3)
Cardiovascular disease	105 (9.7)	992 (9.1)
Myocardial infarction	55 (5.1)	559 (5.1)
Ischemic stroke	29 (2.7)	274 (2.5)
Peripheral artery disease	24 (2.2)	249 (2.3)
Heart failure	40 (3.7)	329 (3.0)
Atrial fibrillation	65 (6.0)	589 (5.4)
Deep vein thrombosis	40 (3.7)	208 (1.9)
Pulmonary embolism	13 (1.2)	103 (0.9)
Chronic obstructive pulmonary disease	32 (2.9)	237 (2.2)
Asthma	100 (9.2)	376 (3.5)
Malignancy	158 (14.5)	1,740 (16.0)
Rheumatoid arthritis	17 (1.6)	96 (0.9)
Systemic inflammatory disease	33 (3.0)	129 (1.2)
Inflammatory bowel disease	17 (1.6)	159 (1.5)

*EU 15 comprises of Austria, Belgium, Denmark, Finland, France, Germany, Greece, Ireland, Italy, Luxembourg, Netherlands, Portugal, Spain, Sweden, United Kingdom. The Nordic countries include Denmark, Finland, Iceland, Norway, and Sweden.

For peer review only

Table 1b. Pharmacological treatments of the study population.

	**Covid-19 (n = 1,086)**	**Control subjects (n = 10,860)**
**Treatments** No. (%)		
Antihypertensive treatments		
ACE inhibitors	168 (15.5)	1,310 (12.1)
ARBs	218 (20.1)	1,694 (15.6)
Calcium-channel blockers	239 (22.0)	1,648 (15.2)
Beta-blockers	222 (20.4)	1,849 (17.0)
Diuretics	51 (4.7)	522 (4.8)
Antidiabetic treatments		
Any antidiabetics	240 (22.1)	1,144 (10.5)
Insulins	80 (7.4)	391 (3.6)
Biguanides	200 (18.4)	855 (7.9)
Sulfonylureas	28 (2.6)	93 (0.9)
Glitazons	6 (0.6)	10 (0.1)
DPP-4 inhibitors	44 (4.1)	210 (1.9)
GLP-1 RAs	37 (3.4)	184 (1.7)
SGLT-2 inhibitors	46 (4.2)	184 (1.7)
Meglitinides	4 (0.4)	34 (0.3)
Statins	288 (26.5)	2,242 (20.6)
Aspirin	136 (12.5)	1,103 (10.2)
Other Antiplatelet drugs	20 (1.8)	234 (2.2)
Warfarin	17 (1.6)	150 (1.4)
NOAC	45 (4.1)	474 (4.4)

Abbreviations: ACE-inhibitors, angiotensin converting enzyme-inhibitors; ARBs, angiotensin receptor blockers; DPP-4 inhibitors, dipeptidyl peptidase-4 inhibitors; GLP-1 RAs, glucagon-like peptide-1 receptor agonists; SGLT2 inhibitors, sodium-glucose cotransporter-2 inhibitors NOAC, new oral anticoagulants

For peer review only

Table 2a. Odds ratios for Covid-19 requiring mechanical ventilation by cardiometabolic factors and other comorbidities..

	Adjusted for age and sex			Adjusted model 2†			Adjusted model 3 ‡		
Risk factors	OR	95% CI	p-value	OR	95% CI	p-value	OR	95% CI	p-value
Type 1 diabetes	2.32	1.12-4.81	0.023	3.13	1.52-6.42	0.002	2.56	1.25-5.24	0.010
Type 2 diabetes	2.73	2.33-3.20	<0.001	2.25	1.90-2.65	<0.001	1.81	1. 49-2.19	<0.001
Obesity	3.23	2.56-4.08	<0.001	3.13	2.43-4.02	<0.001	2.03	1.55-2.65	<0.001
Hypertension	1.76	1.52-2.05	<0.001	1.73	1.48-2.01	<0.001	1.26	1.05--1.51	<0.013
Hyperlipidaemia	1.35	1.15-1.58	<0.001	1.22	1.03-1.43	<0.018	0.90	0.75-1.09	0.286
CKD	2.83	1.97-4.05	<0.001	2.51	1.69-3.70	<0.001	1.84	1.21-2.82	0.005
CVD	1.07	0.86-1.33	0.554	1.03	0.82-1.29	0.789	0.75	0.58-0.96	0.022
Heart failure	1.23	0.87-1.73	0.236	1.13	0.79-1.62	0.48	0.78	0.51-1.19	0.253
Atrial fibrillation	1.11	0.85-1.46	0.43	1.24	0.93-1.64	0.136	1.04	0.75-1.43	0.819
VTE	1.74	1.27-2.39	0.001	1.90	1.37-2.62	<0.001	1.65	1.18-2.31	0.004
COPD	1.37	0.94-1.99	0.105	1.34	0.91-1.97	0.133	0.87	0.56-1.36	0.552
Asthma	2.84	2.26-3.58	<0.001	2.78	2.18-3.53	<0.001	2.25	1.74-2.90	<0.001
Malignancy	0.89	0.74-1.06	0.195	0.97	0.80-1.17	0.725	0.85	0.70-1.04	0.103
Rheumatoid arthritis	1.79	1.06-3.00	0.029	1.86	1.11-3.12	0.019	1.27	0.72-2.23	0.407
Systemic infl. disease	2.65	1.79-3.92	<0.001	2.57	1.71-3.86	<0.001	1.96	1.28-2.99	0.002
Infl. bowel disease	1.07	0.65-1.77	0.792	1.21	0.72-2.03	0.479	0.94	0.54-1.64	0.839

†Adjusted for age, sex, educational level, marital status and region of birth.
‡Adjusted for age, sex, educational level, marital status and region of birth and all diagnoses in table 2a

For peer review only

Abbreviations CVD, cardiovascular disease; VTE, venous thromboembolism; COPD, chronic obstructive pulmonary disease; CKD, chronic kidney disease.

For peer review only

For peer review only - http://bmjopen.bmj.com/site/about/guidelines.xhtml

Table 2b. Odds ratios for Covid-19 requiring mechanical ventilation by pharmacological treatments.

	Adjusted for age and sex			Adjusted model 2†			Adjusted model 3 ‡		
**Treatments**	OR	95% CI	p-value	OR	95% CI	p-value	OR	95% CI	p-value
Antihypertensive treatments									
ACE-inhibitors	1.35	1.13-1.62	0.001	1.35	1.12-1.62	0.797	0.99	0.81-1.22	0.931
ARBs	1.39	1.18-1.64	<0.001	1.47	1.24-1.75	<0.001	1.07	0.88-1.30	0.474
CCBs	1.64	1.39-1.93	<0.001	1.64	1.39-1.93	<0.001	1.25	1.03-1.52	0.025
Beta-blockers	1.28	1.08-1.51	0.004	1.27	1.07-1.51	0.007	0.90	0.73-1.11	0.345
Diuretics	0.98	0.72-1.32	0.870	1.00	0.74-1.36	0.996	0.74	0.53-1.03	0.078
Antidiabetic treatments									
Insulins	2.15	1.67-2.77	<0.001	1.90	1.46-2.47	<0.001	0.85	0.62-1.16	0.305
Biguanides	2.72	2.28-3.24	<0.001	2.26	1.88-2.72	<0.001	1.40	1.01-1.94	0.044
Sulfonylureas	3.08	2.01-4.73	<0.001	2.13	1.33-3.41	0.002	1.17	0.72-1.91	0.530
Glitazons	6.03	2.19-16.6	0.001	5.46	1.98-15.0	0.001	2.86	1.04-7.85	0.042
DPP-4 inhibitors	2.16	1.54-3.01	<0.001	1.77	1.25-2.50	0.001	0.91	0.62-1.33	0.632
GLP-1 RA	2.79	1.92-4.03	<0.001	2.71	1.85-3.97	<0.001	1.24	0.82-1.87	0.312
SGLT-2 inhibitors	2.58	1.85-3.59	<0.001	2.30	1.63-3.25	<0.001	1.20	0.82-1.74	0.346
Meglitinides	1.18	0.42-3.33	0.383	1.03	0.36-2.96	0.953	0.55	0.19-1.61	0.276
Statins	1.44	1.23-1.69	<0.001	1.32	1.12-1.55	0.001	0.84	0.63-1.13	0.247
Aspirin	1.29	1.05-1.57	0.013	1.14	0.93-1.41	0.200	0.97	0.75-1.24	0.791
Warfarin	1.14	0.68-1.90	0.622	1.29	0.76-2.17	0.347	0.96	0.52-1.76	0.894
NOAC	0.95	0.69-1.30	0.730	1.04	0.75-1.44	0.806	0.70	0.46-1.06	0.093

For peer review only

†Adjusted for age, sex, educational level, marital status and region of birth.
‡Adjusted for age, sex, educational level, marital status and region of birth and all diagnoses in table 2a
Abbreviations: ACE-inhibitors, angiotensin converting enzyme-inhibitors; ARBs, angiotensin receptor blockers; CCBs, calcium-channel blockers; DPP-4 inhibitors, dipeptidyl peptidase-4 inhibitors; GLP-1 RA, glucagon-like peptide-1 receptor agonists; SGLT2 inhibitors, sodium-glucose cotransporter-2 inhibitors; NOAC, new oral anticoagulants

For peer review only

Table 3. Covid-19 risk factors by subgroups of age, sex and region of birth. Odds ratios for Covid-19 receiving mechanical ventilation compared to matched control subjects by age (below/over median age 62 years), sex and region of birth (EU15).

	Type 2 diabetes	Obesity	Hypertension	CKD	CVD	VTE	A
**Age**							
>62 years	1.7 (1.3-2.1)	2.2 (1.5-3.4)	1.3 (1.1-1.6)	1.7 (1.0-2.9)	0.9 (0.7-1.2)	1.8 (1.2-2.7)	2.1 (
<62 years	3.3 (2.6-4.2)	4.0 (2.9-5.5)	2.2 (1.8-2.6)	3.1 (1.6-6.1)	1.6 (1.1-2.4)	2.2 (1.3-3.7)	3.7 (
p-value	<0.001	0.027	<0.001	0.010	0.013	0.554	[illegible]
**Sex**							
Male	2.0 (1.7-2.4)	3.1 (2.3-4.3)	1.7 (1.4-2.0)	2.2 (1.4-3.4)	1.0 (0.8-1.3)	1.8 (1.3-2.6)	2.3 (
female	3.5 (2.5-5.0)	3.2 (2.0-5.0)	1.8 (1.3-2.5)	4.2 (1.9-9.3)	1.2 (0.7-2.2)	2.2 (1.1-4.2)	3.9 (
p-value	0.001	0.922	0.327	0.113	0.416	0.558	[illegible]
**Region of birth**							
Outside EU15	3.3 (2.6-4.2)	2.3 (1.6-3.4)	1.8 (1.5-2.3)	2.5 (1.4-4.6)	1.4 (1.0-1.9)	1.4 (0.8-2.4)	3.2 (
Within EU 15/Nordic	2.0 (1.6-2.6)	3.7 (2.7-5.1)	1.6 (1.3-1.9)	2.6 (1.6-4.2)	0.9 (0.7-1.2)	2.0 (1.4-3.0)	2.7 (
p-value	0.004	0.066	0.476	0.872	0.072	0.248	[illegible]

P-values denote likelihood-ratio tests between a model with and one without an interaction term between the indicator variable for the subgroup and the risk factor added to model 2. **Abbreviations:** CKD, chronic kidney disease, CVD, cardiovascular disease (history of myocardial infarction, ischemic stroke or peripheral arterial disease); VTE, venous thromboembolic disease.

For peer review only

For peer review only - http://bmjopen.bmj.com/site/about/guidelines.xhtml

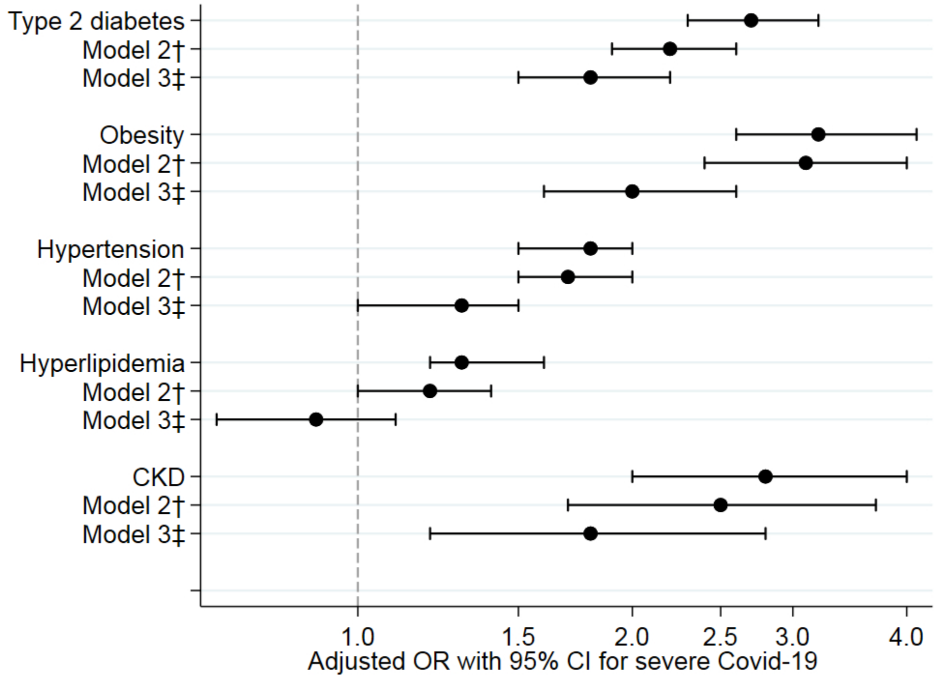

Associations of cardiometabolic risk factors with severe Covid-19 (adjusted odds ratios with 95% Cis).

364x265mm (72 x 72 DPI)

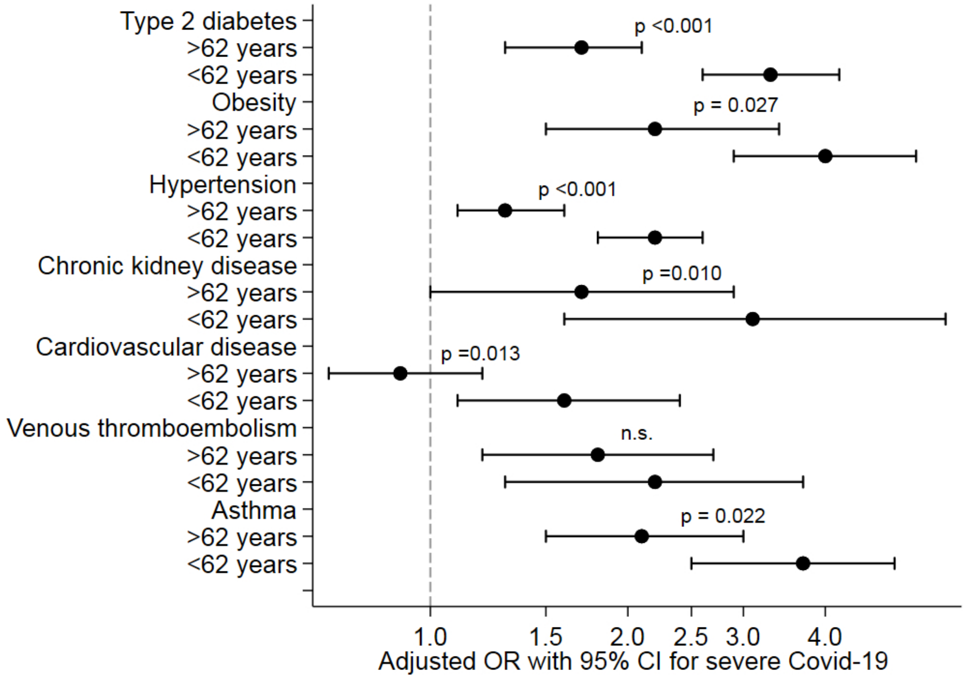

Associations of cardiometabolic risk factors with severe Covid-19 by age below or over median age of 62 years (adjusted odds ratios with 95% Cis).

364x265mm (72 x 72 DPI)

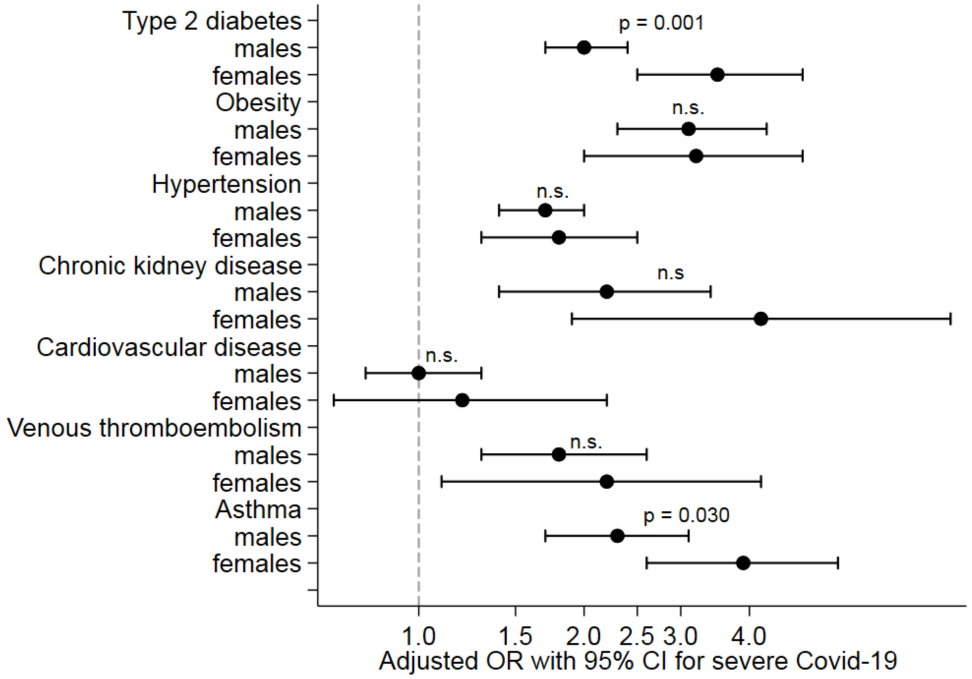

Associations of cardiometabolic risk factors with severe Covid-19 by sex (adjusted odds ratios with 95% Cis).

358x260mm (72 x 72 DPI)

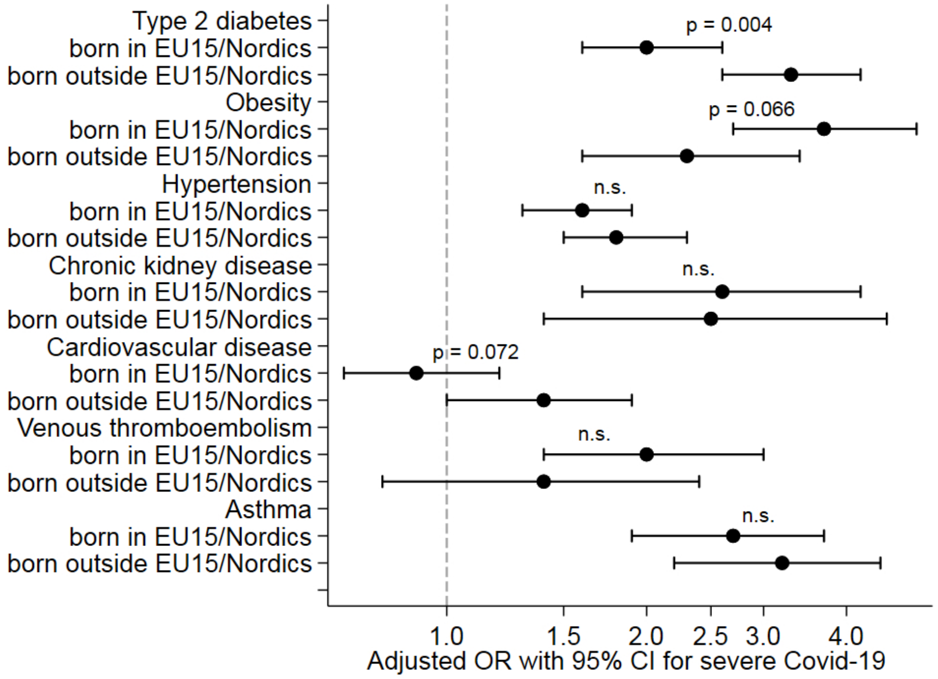

Associations of cardiometabolic risk factors with severe Covid-19 by region of birth * (adjusted odds ratios with 95% Cis).

358x260mm (72 x 72 DPI)

Online Supplementary data

Cardiometabolic factors and risk for severe COVID-19 requiring invasive mechanical ventilation during the Swedish epidemic

Svensson P, Hofmann R, Habel H, Jernberg T, Nordberg P

Table of contents

Supplemental methods – register information 1

eTable 1 – Hypertension definitions .... **Fel! Bokmärket är inte definierat.**

eTable 2 - Missing Data in the Study Population 3

eTable 3 - Baseline characteristics of the study population by age-group 4

eTable 4 - Baseline characteristics of the study population by sex 5

eTable 5 - Baseline characteristics of the study population by region of birth..6

Supplemental figure S1 - Directed Acyclic Graph..7

Supplemental Figure S2 – Exclusion flowchart.. 8

For peer review only

Supplemental methods – register information

The Population Register
The Population Register is managed by Statistics Sweden and includes information on deaths, emigration and immigration for the entire Swedish population. All residents are assigned a unique personal identity number that can be used for linkage of different data resources including several national health registers of high quality.

The longitudinal integrated database for health insurance and labor market studies (LISA)
LISA is managed by Statistics Sweden and includes annual measurements on several socioeconomic and sociodemographic variables, including income, education and country of birth.

The Swedish Patient Register
Swedish Patient Register is managed by the National Board of Health and Welfare and covers inpatient care since 1964- (nationwide since 1987) and non-primary outpatient care since 2001. The register is nationwide with a near complete coverage during the study period.

The Swedish Prescribed Drug Register
The Swedish Prescribed Drug Register is managed by the National Board of Health and Welfare and started on July 1, 2005. The register covers all drugs except over-the-counter medication (which is not covered at all) and medications administered at hospitals (which is only covered to some extent in the Prescribed Drug Register and completely covered through the National Patient Register in some counties).

For peer review only

eTable 1 – Hypertension definition.

**Anti-hypertensive drugs**	**ATC-codes**	**Exclusion diagnosis (ICD-10)**
Diuretics	C03A, C03D, C03E	
Beta-blockers *	C07A, C07F	Angina pectoris (I208, I209)<br>Atrial fibrillation (I48)<br>MI (I21, I22)<br>Heart failure (I50)
Calcium channel blockers	C08C, C08D	
ACE-inhibitors †	C09A, C09B	Heart failure (I50)
Angiotensin receptor blockers ‡	C09C, C09DA, C09DB	Heart failure (I50)
Other drugs targeting blood vessels	C02C, C02D	

Patients with a pick-up of a prescription of the anti-hypertensive drugs within the preceding 12 months of the index date were considered as having hypertension. Beta-blockers, ACE-inhibitors, and Angiotensin receptor blockers may be prescribed for other diagnoses than hypertension. Patients were not classified as having hypertension if these drugs were found in combinations with any such diagnosis. An existing record of hypertension (I109) was superior to the pick-ups of prescribed drugs.

* Patients with a diagnosis of angina pectoris (I208, I209), atrial fibrillation (I48), MI (I21, I22) or heart failure (I50) and simultaneously prescribed with beta-blockers were not classified as having hypertension.

† ‡ Patients with a diagnosis of heart failure (I50) with concurrently prescription of ACE inhibitors or angiotensin receptor blockers were not classified as having hypertension.

For peer review only

eTable 2 - Missing Data[*] in the Study Cohort

Characteristic	Missing data, No. (%)
Age	0 (0.0)
Sex	0 (0.0)
Level of education	239 (2.0)
Region of birth	5 (0.1)
Fills of prescriptions	0 (0)
Medical history	0 (0)

For peer review only

eTable 3. Baseline characteristics of the study population by age-group. Characteristics of patients with Covid-19 requiring mechanical ventilation and control subjects

	**Age below median (62 years)**		**Age above median (62 years)**	
	**Covid-19 (n = 580)**	**Control subjects (n = 5,800)**	**Covid-19 (n = 506)**	**Control subjects (n = 5,600)**
Age, median (IQR), y	53 (47-59)	53 (47-59)	70 (66-75)	70 (66-75)
**Sex:**				
Male, No. (%)	4,230 (72.9)	423 (72.9)	390 (77.1)	3,900 (77.1)
**Sociodemographics** No. (%)				
Education (years)				
≤9	131 (23.5)	886 (15.6)	149 (30.5)	1,258 (25.2)
10-12	258 (46.2)	2,654 (46.7)	208 (42.6)	2,151 (43.2)
≥12	169 (30.3)	2,138 (37.7)	131 (26.8)	1,574 (31.6)
Marital status				
Unmarried	250 (43.1)	3,000 (51.7)	204 (40.3)	2,219 (43.9)
Married	330 (56.9)	2,800 (48.3)	302 (59.7)	2,841 (56.1)
Region of birth				
EU 15* and/or Nordics	271 (46.8)	4,099 (70.7)	325 (64.6)	4,312 (85.2)
**Medical history** No. (%)				
Diabetes mellitus	139 (24.0)	430 (7.4)	146 (28.9)	864 (17.1)
Obesity	68 (11.7)	186 (3.2)	31 (6.1)	142 (2.8)
Hypertension	217 (37.4)	1,269 (21.9)	330 (65.2)	2,989 (59.1)
Hyperlipidaemia	43 (7.4)	182 (3.1)	86 (17.0)	616 (12.2)
Chronic kidney disease	20 (3.4)	40 (0.7)	20 (4.0)	106 (2.1)
Cardiovascular disease	32 (5.5)	202 (3.5)	73 (14.4)	790 (15.6)
Myocardial infarction	23 (4.0)	119 (2.1)	32 (6.3)	440 (8.7)
Ischemic stroke	6 (1.0)	55 (0.9)	23 (4.5)	219 (4.3)
Peripheral artery disease	5 (0.9)	51 (0.9)	19 (3.8)	198 (3.9)
Heart failure	17 (2.9)	57 (1.0)	23 (4.5)	272 (5.4)
Atrial fibrillation	12 (2.1)	93 (1.6)	53 (10.5)	496 (9.8)
Deep vein thrombosis	16 (2.8)	77 (1.3)	24 (4.7)	131 (2.6)
Pulmonary embolism	2 (0.3)	29 (0.5)	24 (4.7)	131 (2.6)
Chronic obstructive pulmonary disease	7 (1.2)	46 (0.8)	25 (4.9)	191 (3.8)
Asthma	57 (9.8)	176 (3.0)	43 (8.5)	200 (4.0)
Malignancy	40 (6.9)	476 (8.2)	118 (23.3)	1,264 (25.0)
Rheumatoid arthritis	8 (1.4)	39 (0.7)	9 (1.8)	57 (1.1)
Systemic inflammatory disease	11 (1.9)	29 (0.5)	22 (4.3)	100 (2.0)
Inflammatory bowel disease	5 (0.9)	81 (1.4)	12 (2.4)	78 (1.5)

* EU 15 comprises of Austria, Belgium, Denmark, Finland, France, Germany, Greece, Ireland, Italy, Luxembourg, Netherlands, Portugal, Spain, Sweden, United Kingdom. The Nordic countries include Denmark, Finland, Iceland, Norway, and Sweden.

For peer review only

eTable 4. Baseline characteristics of the study population by sex. Characteristics of patients with Covid-19 requiring mechanical ventilation and control subjects

	Males		Females	
	**Covid-19 (n = 813)**	**Control subjects (n = 8,130)**	**Covid-19 (n = 273)**	**Control subjects (n = 2,730)**
Age, median (IQR), y	62 (54-70)	62 (54-70)	60 (50-68)	60 (50-68)
**Sociodemographics** No. (%)				
Education (years)				
≤9	204 (25.9)	1,678 (21.0)	76 (29.5)	466 (17.3)
10-12	351 (44.5)	3,606 (45.2)	115 (44.6)	1,199 (44.6)
≥12	233 (29.6)	2,691 (33.7)	67 (26.0)	1,021 (38.0)
Marital status				
Unmarried	314 (38.6)	3,760 (46.2)	140 (51.3)	1,459 (53.4)
Married	499 (61.4)	4,370 (53.8)	133 (48.7)	1,271 (46.6)
Region of birth				
EU 15* and/or Nordics	439 (54.3)	6,250 (76.9)	157 (57.5)	2,161 (79.2)
**Medical history** No. (%)				
Diabetes mellitus	216 (26.6)	1,099 (13.5)	69 (25.3)	195 (7.1)
Obesity	63 (7.7)	218 (2.7)	36 (13.2)	110 (4.0)
Hypertension	419 (51.5)	3,332 (41.0)	128 (46.9)	926 (33.9)
Hyperlipidaemia	103 (12.7)	667 (8.2)	26 (9.5)	131 (4.8)
Chronic kidney disease	26 (3.2)	121 (1.5)	14 (5.1)	25 (0.9)
Cardiovascular disease	91 (11.2)	877 (10.8)	14 (5.1)	115 (4.2)
Myocardial infarction	51 (6.3)	521 (6.4)	4 (1.5)	38 (1.4)
Ischemic stroke	22 (2.7)	225 (2.8)	7 (2.6)	49 (1.8)
Peripheral artery disease	19 (2.3)	216 (2.7)	5 (1.8)	33 (1.2)
Heart failure	30 (3.7)	286 (3.5)	10 (3.7)	43 (1.6)
Atrial fibrillation	53 (6.5)	510 (6.3)	12 (4.4)	79 (2.9)
Deep vein thrombosis	31 (3.8)	163 (2.0)	9 (3.3)	45 (1.6)
Pulmonary embolism	9 (1.1)	81 (1.0)	4 (1.5)	22 (0.8)
Chronic obstructive pulmonary disease	24 (3.0)	184 (2.3)	8 (2.9)	53 (1.9)
Asthma	58 (7.1)	256 (3.1)	42 (15.4)	120 (4.4)
Malignancy	122 (15.0)	1,287 (15.8)	36 (13.2)	453 (16.6)
Rheumatoid arthritis	9 (1.1)	54 (0.7)	8 (2.9)	42 (1.5)
Systemic inflammatory disease	13 (1.6)	64 (0.8)	20 (7.3)	65 (2.4)
Inflammatory bowel disease	12 (1.5)	109 (1.3)	5 (1.8)	50 (1.8)

* EU 15 comprises of Austria, Belgium, Denmark, Finland, France, Germany, Greece, Ireland, Italy, Luxembourg, Netherlands, Portugal, Spain, Sweden, United Kingdom. The Nordic countries include Denmark, Finland, Iceland, Norway, and Sweden.

For peer review only

eTable 5. Baseline characteristics of the study population by region of birth. Characteristics of patients with Covid-19 requiring mechanical ventilation and control subjects

	**Region of birth within EU15/ and/or Nordic Countries**		**Region of birth outside EU15/ and/or Nordic Countries**	
	**Covid-19 (n = 596)**	**Control subjects (n = 5,960)**	**Covid-19 (n = 490)**	**Control subjects (n = 4,900)**
Age, median (IQR), y	64 (54-72)	64 (54-72)	59 (51-67)	59 (51-67)
**Sex:**				
Male, No. (%)	439 (73.7)	4,390 (73.7)	374 (76.3)	3,740 (76.3)
**Sociodemographics** No. (%)				
Education (years)				
≤9	121 (20.6)	1,161 (19.8)	159 (34.6)	983 (20.5)
10-12	295 (50.3)	2,636 (44.9)	171 (37.3)	2,169 (45.2)
≥12	171 (29.1)	2,069 (35.3)	129 (28.1)	1,643 (34.3)
Marital status				
Unmarried	299 (50.2)	2,859 (48.0)	155 (31.6)	2,360 (48.2)
Married	297 (49.8)	3,101 (52.0)	335 (68.4)	2,540 (51.8)
**Medical history** No. (%)				
Diabetes mellitus	128 (21.5)	699 (11.7)	157 (32.0)	595 (12.1)
Obesity	60 (10.1)	170 (2.9)	39 (8.0)	158 (3.2)
Hypertension	313 (52.5)	2,547 (42.7)	234 (47.8)	1,711 (34.9)
Hyperlipidaemia	75 (12.6)	496 (8.3)	54 (11.0)	302 (6.2)
Chronic kidney disease	21 (3.5)	83 (1.4)	19 (3.9)	63 (1.3)
Cardiovascular disease	57 (9.6)	622 (10.4)	48 (9.8)	370 (7.6)
Myocardial infarction	29 (4.9)	338 (5.7)	26 (5.3)	221 (4.5)
Ischemic stroke	16 (2.7)	174 (2.9)	13 (2.7)	100 (2.0)
Peripheral artery disease	15 (2.5)	158 (2.7)	9 (1.8)	91 (1.9)
Heart failure	21 (3.5)	192 (3.2)	19 (3.9)	137 (2.8)
Atrial fibrillation	53 (8.9)	373 (6.3)	12 (2.4)	216 (4.4)
Deep vein thrombosis	25 (4.2)	112 (1.9)	15 (3.1)	96 (2.0)
Pulmonary embolism	8 (1.3)	64 (1.1)	5 (1.0)	39 (0.8)
Chronic obstructive pulmonary disease	18 (3.0)	125 (2.1)	14 (2.9)	112 (2.3)
Asthma	53 (8.9)	212 (3.6)	47 (9.6)	164 (3.3)
Malignancy	105 (17.6)	1,075 (18.0)	53 (10.8)	665 (13.6)
Rheumatoid arthritis	9 (1.5)	64 (1.1)	8 (1.6)	32 (0.7)
Systemic inflammatory disease	21 (3.5)	77 (1.3)	12 (2.4)	52 (1.1)
Inflammatory bowel disease	14 (2.3)	89 (1.5)	3 (0.6)	70 (1.4)

* EU 15 comprises of Austria, Belgium, Denmark, Finland, France, Germany, Greece, Ireland, Italy, Luxembourg, Netherlands, Portugal, Spain, Sweden, United Kingdom. The Nordic countries include Denmark, Finland, Iceland, Norway, and Sweden.

For peer review only - http://bmjopen.bmj.com/site/about/guidelines.xhtml

For peer review only

Supplemental Fig. S1 – Directed Acyclic Graph

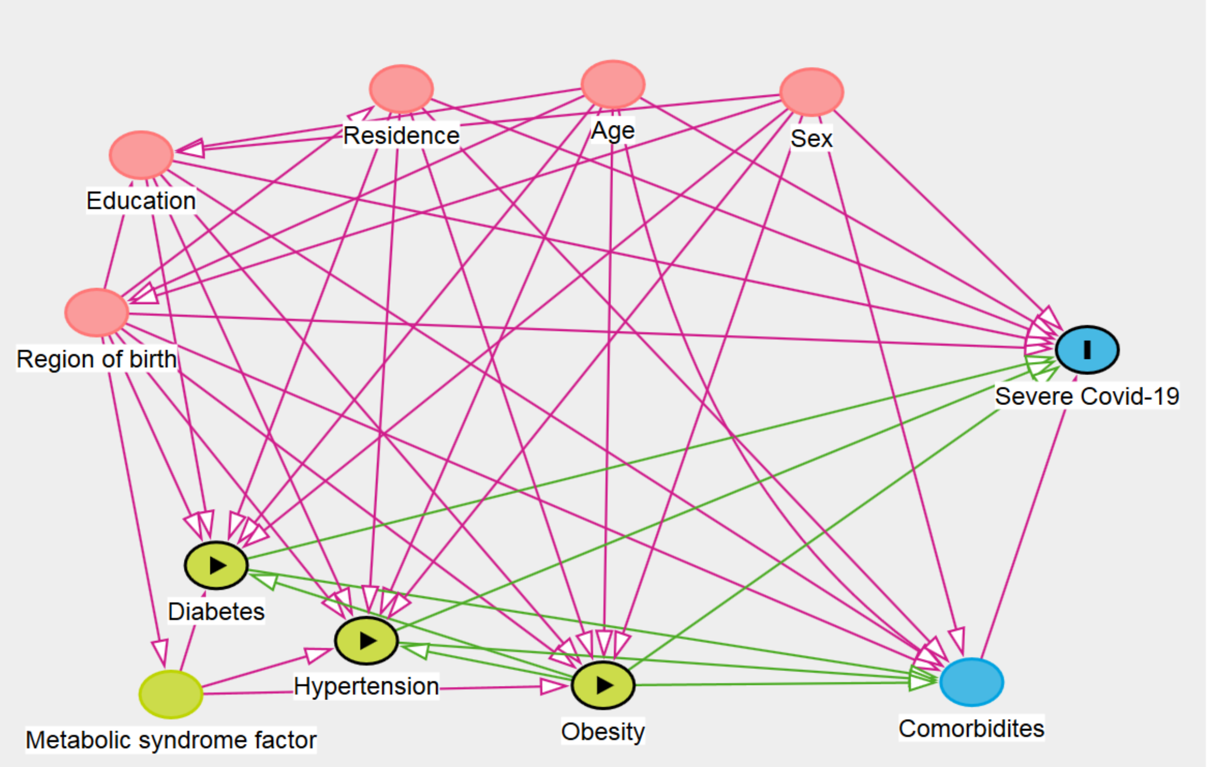

For peer review only

Supplemental Fig. S2 - Flow chart of exclusion criteria

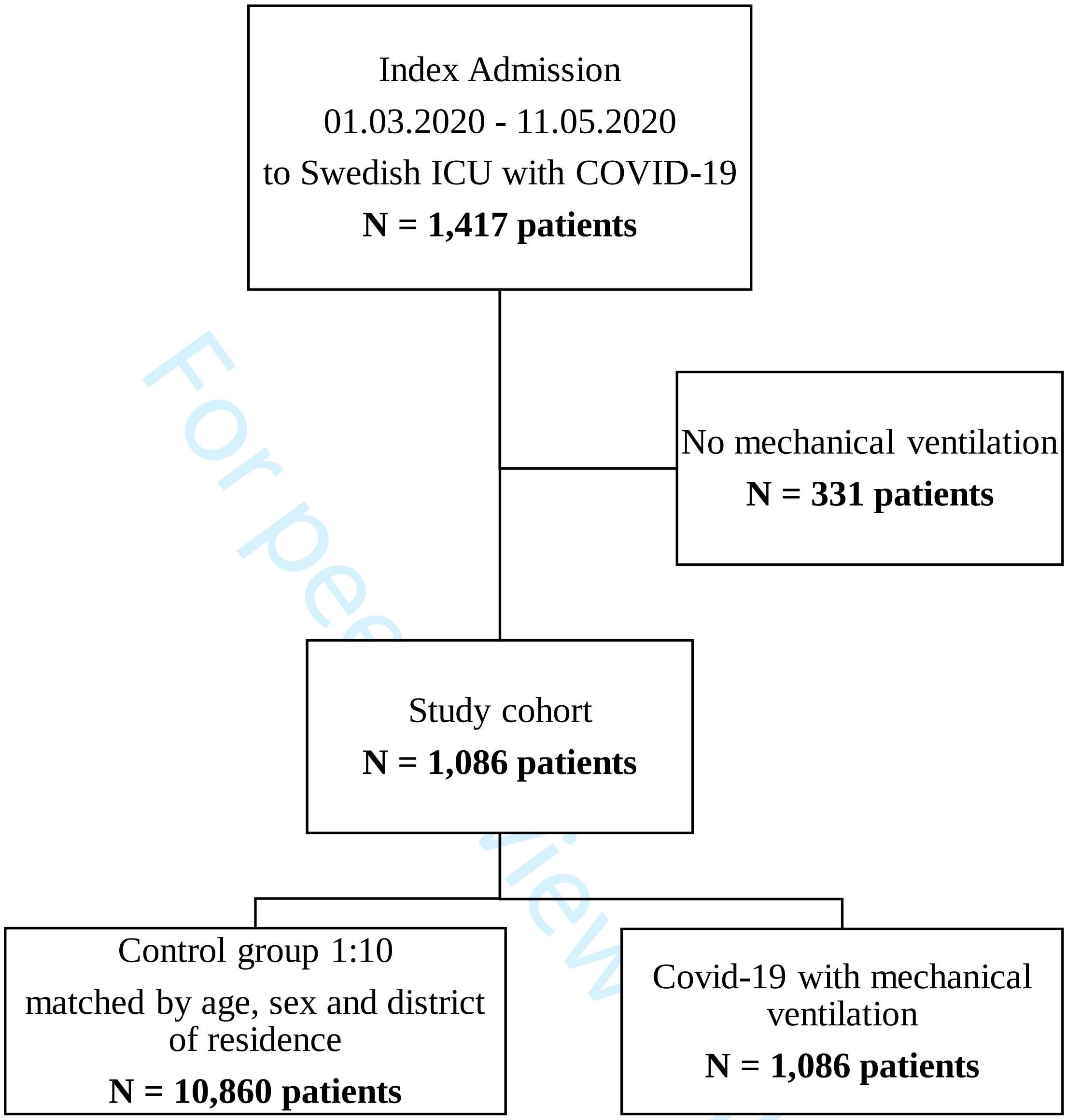

For peer review only

For peer review only - http://bmjopen.bmj.com/site/about/guidelines.xhtml

STROBE Statement—Checklist of items that should be included in reports of ***case-control studies***

	Item No	Recommendation	Page No
**Title and abstract**	1	(*a*) Indicate the study's design with a commonly used term in the title or the abstract	2
		(*b*) Provide in the abstract an informative and balanced summary of what was done and what was found	2
**Introduction**			
Background/rationale	2	Explain the scientific background and rationale for the investigation being reported	5
Objectives	3	State specific objectives, including any prespecified hypotheses	6
**Methods**			
Study design	4	Present key elements of study design early in the paper	6
Setting	5	Describe the setting, locations, and relevant dates, including periods of recruitment, exposure, follow-up, and data collection	6
Participants	6	(*a*) Give the eligibility criteria, and the sources and methods of case ascertainment and control selection. Give the rationale for the choice of cases and controls	6
		(*b*) For matched studies, give matching criteria and the number of controls per case	6
Variables	7	Clearly define all outcomes, exposures, predictors, potential confounders, and effect modifiers. Give diagnostic criteria, if applicable	7,8
Data sources/ measurement	8*	For each variable of interest, give sources of data and details of methods of assessment (measurement). Describe comparability of assessment methods if there is more than one group	7
Bias	9	Describe any efforts to address potential sources of bias	9
Study size	10	Explain how the study size was arrived at	6
Quantitative variables	11	Explain how quantitative variables were handled in the analyses. If applicable, describe which groupings were chosen and why	8
Statistical methods	12	(*a*) Describe all statistical methods, including those used to control for confounding	8
		(*b*) Describe any methods used to examine subgroups and interactions	8
		(*c*) Explain how missing data were addressed	8
		(*d*) If applicable, explain how matching of cases and controls was addressed	8
		(*e*) Describe any sensitivity analyses	
**Results**			
Participants	13*	(a) Report numbers of individuals at each stage of study—eg numbers potentially eligible, examined for eligibility, confirmed eligible, included in the study, completing follow-up, and analysed	Suppl Figure S2
		(b) Give reasons for non-participation at each stage	Suppl Figure S2
		(c) Consider use of a flow diagram	Suppl Figure S2
Descriptive data	14*	(a) Give characteristics of study participants (eg demographic, clinical, social) and information on exposures and potential confounders	9

For peer review only - http://bmjopen.bmj.com/site/about/guidelines.xhtml

		(b) Indicate number of participants with missing data for each variable of interest	Etable2
Outcome data	15*	Report numbers in each exposure category, or summary measures of exposure	Table 2

For peer review only

For peer review only - http://bmjopen.bmj.com/site/about/guidelines.xhtml

Main results	16	(*a*) Give unadjusted estimates and, if applicable, confounder-adjusted estimates and their precision (eg, 95% confidence interval). Make clear which confounders were adjusted for and why they were included	Table 3
		(*b*) Report category boundaries when continuous variables were categorized	n.a
		(*c*) If relevant, consider translating estimates of relative risk into absolute risk for a meaningful time period	n.a
Other analyses	17	Report other analyses done—eg analyses of subgroups and interactions, and sensitivity analyses	10
**Discussion**			
Key results	18	Summarise key results with reference to study objectives	13
Limitations	19	Discuss limitations of the study, taking into account sources of potential bias or imprecision. Discuss both direction and magnitude of any potential bias	15
Interpretation	20	Give a cautious overall interpretation of results considering objectives, limitations, multiplicity of analyses, results from similar studies, and other relevant evidence	16
Generalisability	21	Discuss the generalisability (external validity) of the study results	15
**Other information**			
Funding	22	Give the source of funding and the role of the funders for the present study and, if applicable, for the original study on which the present article is based	17

*Give information separately for cases and controls.

Note: An Explanation and Elaboration article discusses each checklist item and gives methodological background and published examples of transparent reporting. The STROBE checklist is best used in conjunction with this article (freely available on the Web sites of PLoS Medicine at http://www.plosmedicine.org/, Annals of Internal Medicine at http://www.annals.org/, and Epidemiology at http://www.epidem.com/). Information on the STROBE Initiative is available at http://www.strobe-statement.org.

For peer review only

BMJ Open

Association between cardiometabolic disease and severe Covid-19: a nationwide case-control study of patients requiring invasive mechanical ventilation

Journal:	*BMJ Open*
Manuscript ID	bmjopen-2020-044486.R1
Article Type:	Original research
Date Submitted by the Author:	19-Dec-2020
Complete List of Authors:	Svensson, Per; Karolinska Institutet Department of Clinical Science and Education Sodersjukhuset,<br>Hofmann, Robin; Karolinska Institute, Department of Clinical Science and Education, Södersjukhuset<br>Häbel, Henrike; Institute of Environmental Medicine<br>Jernberg, Tomas; Danderyd University Hospital, Karolinska Institutet<br>Nordberg, Per; Karolinska institutet, Dept. of Cardiology, Centre for Reuscitation Science
**Primary Subject Heading**:	Infectious diseases
Secondary Subject Heading:	Cardiovascular medicine, Diabetes and endocrinology, Intensive care
Keywords:	COVID-19, Diabetes & endocrinology < INTERNAL MEDICINE, Hypertension < CARDIOLOGY, INTENSIVE & CRITICAL CARE

SCHOLARONE™
Manuscripts

For peer review only - http://bmjopen.bmj.com/site/about/guidelines.xhtml

I, the Submitting Author has the right to grant and does grant on behalf of all authors of the Work (as defined in the below author licence), an exclusive licence and/or a non-exclusive licence for contributions from authors who are: i) UK Crown employees; ii) where BMJ has agreed a CC-BY licence shall apply, and/or iii) in accordance with the terms applicable for US Federal Government officers or employees acting as part of their official duties; on a worldwide, perpetual, irrevocable, royalty-free basis to BMJ Publishing Group Ltd ("BMJ") its licensees and where the relevant Journal is co-owned by BMJ to the co-owners of the Journal, to publish the Work in this journal and any other BMJ products and to exploit all rights, as set out in our licence.

The Submitting Author accepts and understands that any supply made under these terms is made by BMJ to the Submitting Author unless you are acting as an employee on behalf of your employer or a postgraduate student of an affiliated institution which is paying any applicable article publishing charge ("APC") for Open Access articles. Where the Submitting Author wishes to make the Work available on an Open Access basis (and intends to pay the relevant APC), the terms of reuse of such Open Access shall be governed by a Creative Commons licence – details of these licences and which Creative Commons licence will apply to this Work are set out in our licence referred to above.

Other than as permitted in any relevant BMJ Author's Self Archiving Policies, I confirm this Work has not been accepted for publication elsewhere, is not being considered for publication elsewhere and does not duplicate material already published. I confirm all authors consent to publication of this Work and authorise the granting of this licence.

For peer review only

Association between cardiometabolic disease and severe Covid-19: a nationwide case-control study of patients requiring invasive mechanical ventilation

Per Svensson, MD, PhD[1,2]; Robin Hofmann, MD, PhD[1,2]; Henrike Häbel, PhD[3]; Tomas Jernberg, MD, PhD [4]; and Per Nordberg, MD, PhD[5,2]

1. Department of Clinical Science and Education, Södersjukhuset, Karolinska Institutet, Stockholm, Sweden
2. Department of Cardiology, Södersjukhuset, Stockholm, Sweden
3. Institute of Environmental Medicine, Division of Biostatistics, Karolinska Institutet, Stockholm, Sweden
4. Department of Clinical Sciences, Danderyd University Hospital, Karolinska Institutet, Stockholm, Sweden
5. Department of Medicine Solna, Karolinska Institutet, Stockholm, Sweden

Word count: 4624

Correspondence to: Per Svensson
Assoc. Prof, MD, FESC
Department of Clinical Science and Education, Södersjukhuset, Karolinska Institutet
Department of Cardiology, Södersjukhuset
Sjukhusbacken 10, SE 118 83 Stockholm, Sweden. +46 (0)8 616 3014
Email: per.svensson@ki.se
Twitter: @perpersvensson

For peer review only - http://bmjopen.bmj.com/site/about/guidelines.xhtml

ABSTRACT

Aims:

The risks associated with diabetes, obesity and hypertension for severe Covid-19 may be confounded and differ by sociodemographic background. We assessed the risks associated with cardiometabolic factors for severe Covid-19 when accounting for socioeconomic factors and in subgroups by age, sex and region of birth.

Methods and results

In this nationwide case-control study, 1.086 patients admitted to intensive care with Covid-19 requiring mechanical ventilation (cases) and 10.860 population-based controls matched for age, sex and district of residency were included from mandatory national registries. Odds Ratios (ORs) with 95% confidence intervals (CIs) for associations between severe Covid-19 and exposures with adjustment for confounders were estimated using logistic regression. The median age was 62 years (IQR 52-70), and 3,003 (24.9%) were female. Type 2 diabetes (OR, 2.3 [95% CI, 1.9-2.7]), hypertension (OR, 1.7 [95% CI, 1.5-2.0]), obesity (OR, 3.1 [95% CI, 2.4-4.0]) and chronic kidney disease (OR, 2.5 [95% CI, 1.7-3.7]) were all associated with severe Covid-19. In the younger subgroup (below 57 years) ORs were significantly higher for all cardiometabolic risk factors. The risk associated with type 2 diabetes was higher in women (p=0.001) and in patients with a region of birth outside EU (p=0.004).

Conclusion

Diabetes, obesity and hypertension were all independently associated with severe Covid-19 with stronger associations in the younger population. Type 2 diabetes implied a greater risk among women and in non-EU immigrants. These findings, originating from high-quality

For peer review only

Swedish registries, may be important to direct preventive measures such as vaccination to susceptible patient groups.

Trial registration: Clinicaltrial.gov (NCT04426084)

For peer review only

For peer review only - http://bmjopen.bmj.com/site/about/guidelines.xhtml

STRENGTH AND LIMITATIONS OF THIS STUDY

- In contrast to many previous reports, this study accounts for severity of Covid-19 and provides a homogeneous study group, by only including intubated patients at the intensive care with the highest risk of death. By inclusion of virtually all such cases nationwide, in a country with a tax-financed health care and a serious epidemic during the study period, the study provides a large sample size with adequate power and high external validity.
- This study compared severe Covid-19 patients with matched controls in the underlying population and used 10 population-based, age, sex and district of residence matched controls per each case. Therefore, the study can provide estimates of relative risk for severe Covid-19 in the population for the studied risk factors.
- Socioeconomic factors have been crucial in how the pandemic has impacted on different groups in the society but are also closely linked with obesity, diabetes and cardiovascular disease. By matching for district of residence and adjusting for individual level data on several socioeconomic variables this study confirms previous studies but also provides novel evidence that diabetes, obesity and hypertension are independently associated with severe Covid-19, and how the relative importance of risk factors differs by age, sex and region of birth.
- The data on exposures are from high quality national registries and the patient cohort is the most complete Swedish cohort on severe Covid-19 published to date. The findings may be important to direct preventive measures such as vaccination to susceptible patient groups.
- A possible limitation is that the outcome did not include patients where admission to intensive care and/or mechanical ventilation was not considered appropriate and those

For peer review only

who died before intensive care, another limitation is the lack of information on smoking status.

For peer review only

For peer review only - http://bmjopen.bmj.com/site/about/guidelines.xhtml

INTRODUCTION

Observational data suggest both a higher prevalence, and a more severe course of corona virus disease 2019 (Covid-19) among individuals with diabetes[1,2], obesity[3] and hypertension,[4-8] risk factors that are closely linked and cluster together in the metabolic syndrome.[9] As more clinical data are emerging, new determinants of both Covid-19 and severity of disease are being discussed, such as coagulation disorders[10] and socio-economic factors[11] but to this point the underlying mechanisms that link cardiometabolic disease with severe Covid-19 remain unclear.[12]

In order to advance the knowledge on risk factors, several aspects are crucial to put evidence into perspective: First, the spectrum of disease severity needs to be addressed as the clinical presentation of infected patients can range from asymptomatic, to severe with high risk of fatal outcome. As the risks of being infected may differ from the risk of becoming severely ill once infected, there is a need for studies that focus on risk factors associated with a severe disease. Second, as socio-economic and cultural factors are closely linked to type 2 diabetes, obesity and cardiovascular disease, these need to be accounted for in such analyses. And, most importantly, prevalent cases need to be compared to controls to reliably assess the magnitude of the major risk factors in the underlying population.[13] Currently only a few population-based studies have investigated the association between cardiometabolic risk factors and covid-19 death[1,2,14]. To our knowledge, no study has investigated whether cardiometabolic risk factors are independently associated with severe Covid-19 requiring intensive care, when controlled for age, sex, sociodemographic factors, and immigrant background using matched population-based controls. In addition, it is unknown whether the impact of these risk factors is attenuated by age, sex and sociodemographic factors.

For peer review only - http://bmjopen.bmj.com/site/about/guidelines.xhtml

Sweden has been hit hard by the Covid-19 epidemic but in contrast to most other countries, did not employ a strict lock-down policy. To continuously evaluate the situation, strong governmental efforts were enforced on national health care registries for data reporting.
In this study we present a comprehensive Swedish sample originating from mandatory, high-quality national registries with the aim to investigate whether cardiometabolic risk factors are associated with severe Covid-19 in patients treated at the intensive care unit with invasive mechanical ventilation.

METHODS

Study design and ethics

This nationwide case-control study was based on data from the Swedish Intensive Care Registry (SIR) on patients (cases) with severe Covid-19 admitted to the ICU requiring invasive mechanical ventilation between 1st of March until 11th of May 2020. For each case, 10 controls were randomly selected from the Swedish Population Register and matched by age, sex and district of residence (corresponding to part of municipality). The study database was merged with multiple mandatory Swedish national registries at Statistics Sweden and the National Board of Health and Welfare, which are further described in supplemental methods, using each individual's unique personal identification number.[15] The study complied with the Declaration of Helsinki, was approved by the National Ethical Review Board (identification number 2020/124-31/4) and registered at Clinicaltrial.gov (NCT04426084). The study used already collected, pseudonymized data and involved minimal infringement of personal integrity.

Patient and Public Involvement

For peer review only - http://bmjopen.bmj.com/site/about/guidelines.xhtml

Patients and the public were not involved in the design or conduct of our research.

National registries and Data collection

Severe COVID-19 was defined as laboratory confirmed COVID-19 infection in individuals treated at the intensive care unit (ICU) with mechanical ventilation. These cases were reported to SIR[16] which is a national register with about 95% coverage of all ICU admissions in Sweden and was used to identify eligible patients. The National Patient Register[17] was used to collect primary or secondary diagnoses from previous hospital admissions and outpatients' visits coded according to the International Classification of Diseases (ICD) version 10 within the 15 years preceding the admission. The Prescribed Drugs Register contains information on all dispensed drugs according to the Anatomical Therapeutic Chemical Classification (ATC). We collected individual data on dispensed drugs prescribed and claimed within 12 months before the study period. The longitudinal integrated database for health insurance and labor market studies is managed by Statistics Sweden and includes annual measurements on several socioeconomic and sociodemographic variables, including income, education and country of birth.[18]

Definition of Exposures

Exposures were a history of cardiometabolic or relevant chronic disease based on diagnoses in the National Patient Register within 15 years preceding the admission or prescribed drugs within the preceding 12 months. Hypertension (defined as previous diagnosis of ICD I10 or prescription of antihypertensive drugs as described previously[19]), hyperlipidemia (ICD E78 or prescription of lipid lowering drugs), diabetes mellitus type 2(ICD E11 or prescription of antidiabetic drugs), diabetes mellitus type 1 (ICD E10), obesity (ICD E66), heart failure (ICD I50.1, I50.9), atrial fibrillation (ICD I48), venous thromboembolism (ICD I26, I80), asthma

For peer review only

(ICD J45), chronic obstructive pulmonary disease (ICD J44), chronic kidney disease (ICD N18), malignancy (ICD C, D40-48), rheumatoid arthritis (ICD M05, M06), systemic inflammatory disease (ICD M30-M36), and inflammatory bowel disease (ICD K50, K51) were included. A history of cardiovascular disease (CVD) was defined as a record of either MI (ICD I21, I22), ischemic heart disease (ICD I25), ischemic stroke (ICD I63), or peripheral vascular disease (ICD I70-I73), in the Swedish Patient Register (Supplementary eTable1).

Definition of covariates and variables for subgroup analyses

Level of education was categorized as ≤9 years (reference), 10-12 years, and >12 years based on the highest educational level attained during the year before admission. Region of birth was categorized as a country of birth within EU15 (Austria, Belgium, Denmark, Finland, France, Germany, Greece, Ireland, Italy, Luxembourg, Netherlands, Portugal, Spain, Sweden, United Kingdom) and/or the Nordic Countries (Denmark, Finland, Iceland, Norway, Sweden), or having a country of birth outside this region. Marital status during the year before index date was categorized as married or not married which included unmarried, divorced, and widowed. Subgroup analyses were performed for region of birth, sex (male/female) and age tertiles.

Outcome

The outcome was defined as an ICU admission due to Covid-19 (with a laboratory confirmed Sars-Cov2 infection), registered in SIR, with at least one episode of invasive mechanical ventilation during the ICU stay. All eligible patients during the study period between 1st of March until 11th of May 2020 were included as cases in the study. In a sensitivity analysis the outcome was defined as any ICU admission due to Covid-19 (with a laboratory confirmed Sars-Cov2 infection), registered in SIR during the study period.

For peer review only

Statistical Methods

Categorical variables are reported as frequencies and percentages, while continuous variables are reported as median and interquartile range (IQR). Missing data are reported in Supplemental eTable 2. Odds ratios (OR) and 95% confidence intervals (CI) for the association between the different exposures and the outcome were calculated by means of logistic regression adjusted for age and sex (Model 1). For all exposures additional adjustments were made for sociodemographic and socioeconomic variables (marital status, region of birth and educational level) (Model 2) and, finally, for all conditions listed in the paragraph exposures above that were used as covariates in a fully adjusted regression model (Model 3) in order to analyse both total effects unconfounded of sociodemographic variables and direct effects in accordance to our perception of causal relationships as illustrated by the directed acyclic graphs in supplementary figure S1. Standard errors were calculated using the robust sandwich estimator and the significance level was set at an alpha of 0.05. For a formal test of a significant difference between the ORs for different subgroups, likelihood-ratio tests were conducted between a model with and without an interaction term between the indicator variable for the subgroup and the risk factor. For these tests, the robust sandwich estimator was not used in the underlying logistic regression models.

Statistical analyses were performed using Stata version 16.0 (StataCorp, College Station, TX).

For peer review only

RESULTS

During the study period between 1st of March until 11th of May 2020, a total of 1,417 patients were admitted to an intensive care unit in Sweden due to Covid-19 out of which 1,086 required treatment with invasive mechanical ventilation (cases). For each case, 10 matched control subjects were randomly selected, rendering a total of 10,860 control subjects. The study population selection procedure and reasons for exclusions are described in Supplemental Figure S2.

Patient characteristics

The median age was 62 (IQR 52-70) years and 75% were men. Baseline characteristics are summarized in Table 1a. Patients were less likely to have a post-secondary education and more likely to be married compared to the control group. Further, patients were more likely to have history of migration with more patients having a region of birth outside EU15 and the Nordic countries. Comorbid conditions were more common among patients with Covid-19 receiving mechanical ventilation compared to the control group. In particular, cardiometabolic risk factors were overrepresented with more patients having a history of hypertension, hyperlipidemia, diabetes mellitus, obesity and chronic kidney disease, but also venous thromboembolic disease, asthma and systemic inflammatory diseases were more common. Due to more comorbid conditions, patients had correspondingly more pharmacological treatments (table 1b). All antihypertensive treatments were more common among patients compared to controls subjects as were all antidiabetic treatments except meglitinides.

Comparison of risk factors and treatments between patient with severe Covid-19 and controls

For peer review only - http://bmjopen.bmj.com/site/about/guidelines.xhtml

In the multivariable logistic regression models presented in table 2a, both type 2 diabetes (OR, 2.7 [95% CI, 2.3-3.2]), hypertension (OR, 1.8 [95% CI, 1.5-2.0]), hyperlipidemia (OR, 1.4 [95% CI, 1.2-1.6]), obesity (OR, 3.2 [95% CI, 2.6-4.1]) and chronic kidney disease (OR, 2.8 [95% CI, 2.0-4.0]) were associated with Covid-19 receiving mechanical ventilation. All associations remained significant after adjustment for possible socioeconomic confounders. In the fully adjusted model, all cardiometabolic risk factors except hyperlipidemia were associated with the outcome indicating direct and additive effects for these risk factors (figure 1). In addition, we observed associations between a history of venous thromboembolic disease (OR, 1.7 [95% CI, 1.3-2.4]), asthma (OR, 2.8 [95% CI, 2.3-3.6]), rheumatoid arthritis (OR, 1.8 [95% CI, 1.1-3.0]), as well as systemic inflammatory disease (OR, 2.6 [95% CI, 1.8-3.9]) and severe Covid-19. In contrast, neither cardiovascular disease, heart failure, atrial fibrillation, malignancy, chronic obstructive pulmonary disease or inflammatory bowel disease were associated with the outcome.

In the logistic regression analysis adjusted for age and sex using no treatment as reference, all types of antihypertensive treatment, except diuretics and all types of antidiabetic treatments, except meglinitides, were associated with severe Covid-19 (table 2b). However, when adjusting for all comorbidities in the fully adjusted model, calcium-channel blockers, biguanides and glitazones were the only treatments which remained associated with the outcome.

Subgroup analysis

Baseline characteristics by subgroups of age, sex and region of birth are summarized in supplemental eTable 3, eTable 4 and eTable 5 respectively. A regression analysis for the cardiometabolic risk factors is presented in table 3 by subgroups of age(tertiles), sex and region of birth. In the younger subgroup (aged 21-56 years), odds ratios were significantly

For peer review only - http://bmjopen.bmj.com/site/about/guidelines.xhtml
For peer review only

higher for hypertension (OR, 2.6 [95% CI, 2.0-3.3]), type 2 diabetes (OR, 4.5 [95% CI, 3.3-6,2]), obesity (OR, 7.6 [95% CI, 5.3-11.0]), chronic kidney disease (OR, 7.7 [95% CI, 3.4-17.5]) (p=0.010), venous thromboembolic disease (OR, 3.9 [95% CI, 2.0-7.6]), asthma (OR, 4.9 [95% CI, 3.3-7.3]), systemic inflammatory disease (OR, 6.7[95% CI, 3.0-14.9]) and heart failure (OR, 5.4[95% CI, 2.4-12.2]) as illustrated in figure 2. In women, the odds ratios for type 2 diabetes and asthma were significantly higher as compared to men (figure 3). Among patients with a region of birth outside EU 15, diabetes had a stronger association with severe Covid-19 compared to patients with a region of birth within EU15, whereas a trend towards the opposite was observed for obesity (figure 4).

Sensitivity analysis

In a sensitivity analysis, we report any Covid-19 related ICU-admission (with or without mechanical ventilation) as the outcome (eTable 6) which was found in a total of 1417 patients. Associations with cardiometabolic risk factors were similar but positive associations were also observed with heart failure (OR, 1.6 [95% CI, 1,2-2.1]), atrial fibrillation (OR, 1.5 [95% CI, 1.2-1.9]) and chronic obstructive pulmonary disease (OR, 1.9 [95% CI, 1.4-2.5])

For peer review only

DISCUSSION

In the present nationwide case-control study, assessing the risk for severe Covid-19 with need for mechanical ventilation at the intensive care unit, we found that the cardiometabolic risk factors diabetes, obesity and hypertension, were strongly and independently associated with severe infection also when accounting for socioeconomic factors. Furthermore, we found higher risks associated with all cardiometabolic risk factors among younger patients whereas diabetes was more important in women and in those with an immigrant background. These findings, originating from high-quality national registries in Sweden which has been experiencing a serious epidemic, confirm and extend findings from previous studies and may be important to identify susceptible patient groups requiring extra precautions and prioritized for vaccination.

Cardiometabolic risk factors were early linked with a severe Covid-19 in case-series[20] and uncontrolled studies[21] ,later in studies using population-based control subjects.[5] and nationwide studies based on data from electronic health records[1,2,14]. Here, we confirm these findings and extend them in a well-controlled design and can reinforce that diabetes, obesity and hypertension - risk factors that are closely linked and often cluster together in the metabolic syndrome[22] - all have strong and independent direct associations with the outcome. By adjusting for sociodemographic factors collected on individual level, we can also show that obesity and other components of the metabolic syndrome - factors that are closely linked with lower socioeconomic status[23] - act on Covid-19 independent of sociodemography. The effect of diabetes was even stronger in the population with immigrant background.

Initial studies on hypertension as a risk factor for severe disease[24] may have been confounded by age and until now, there has been limited evidence of hypertension being an independent

For peer review only - http://bmjopen.bmj.com/site/about/guidelines.xhtml

risk factor.[25] In a large nationwide study from the UK on risk factors for death in Covid-19,[1] hypertension was positively associated with the outcome when adjusted for age and sex, but it was no longer a risk factor when other comorbid conditions were accounted in the fully adjusted model. In another large study in patient with type 2 diabetes, antihypertensive treatment was independently associated with Covid-19 related mortality.[14] Here, we can report that hypertension is not only a risk factor independent of age and other related conditions, but also that risk factor patterns differ by age, sex, and region of birth. In the younger subgroup (age below 57 years) all cardiometabolic factors had an even stronger association with severe Covid-19. In women and individuals born outside EU 15, diabetes had the strongest association with the outcome. All components of metabolic syndrome are associated with endothelial dysfunction[26] and low-grade inflammation.[27] Hypertension is also linked with a dysregulated immune system,[28] including endothelial mechanisms,[29] and is causally associated with increased lymphocyte count.[30] As emerging evidence suggests that endothelial inflammation is involved in serious manifestations of Covid-19,[31] it is possible that a common mechanism linking cardiometabolic risk factors with severe Covid-19 is mediated through endothelial and microcirculatory dysfunction. Our findings suggest that these mechanisms are even more important at a younger age. Therefore, a more detailed metabolic phenotyping that includes biomarkers of subclinical inflammation and insulin resistance may be important to identify younger patients at the highest risk and further studies are warranted in this field.[3]

We identified asthma, previous thromboembolic disease, rheumatoid arthritis, and systemic inflammatory disease as additional chronic diseases with increased risk for a severe course of Covid-19. This is new and important information as patients with chronic inflammatory conditions may also be more susceptible to the proinflammatory pathways of the infection

For peer review only - http://bmjopen.bmj.com/site/about/guidelines.xhtml

that involves diffuse endothelial inflammation and systemic impaired microcirculation leading to multiorgan dysfunction.[27]

The association between socioeconomic factors and cardiovascular disease is well established[23] and socioeconomic factors have also been important during the Covid-19-pandemic.[32 33] We therefore believe that matching for residency, which is linked with socioeconomic factors, as well as adjustments for individual level information on migration, level of education and marital status, is a crucial factor in our study design.

In comparison to several previous descriptive studies, we have a well-characterised, homogenous, nationwide population with severe disease, all needing mechanical ventilation at the intensive care unit. To the best of our knowledge, this is the first study that includes virtually all cases with this type of severe disease nationwide together with a population based matched control group. In addition, some previous studies include a heterogeneous mix of cases where other factors such as testing patterns in mild cases may have influenced overall results.[5] Consequently, the current study estimated relative risks in the population for developing severe Covid-19 which may differ from the risk of obtaining an infection with a milder course of disease. Since Swedish health care is virtually fully tax funded, all acute treatments including admission to the ICU with invasive mechanical ventilation, are based on medical decisions and do not involve private-economic considerations. Unequal access to health care is thereby reduced, increasing the validity of our results. In addition, vast resources were put to scale up ICU-resources due to the pandemic which resulted in a nationwide surplus of ICU-beds during the study period. However, there are some limitations with the present study. It is possible that intensive care treatment with mechanical ventilation was not considered appropriate in some patients with multiple comorbidities or severe frailty,

For peer review only - http://bmjopen.bmj.com/site/about/guidelines.xhtml

thus our results may underestimate associations with severe Covid-19 for these conditions. Although the indication for early mechanical ventilation in severe Covid-19 has changed somewhat as the pandemic evolved, we think this will have limited impact on our results since we only included the first two months of the first wave in which the indication was rather stable. Further, in terms of external validity, patient selection for intensive care treatment including invasive ventilation may differ between countries,[34] however, it is unlikely that this will affect the relative importance of risk factors. We did not have any information on smoking or concerning the rate of mild infection with Covid-19 among control subjects. Finally, the observational design of the study cannot exclude the potential of residual confounding and the results should be interpreted as such.

For peer review only

For peer review only - http://bmjopen.bmj.com/site/about/guidelines.xhtml

CONCLUSIONS

Diabetes, obesity and hypertension were all independently associated with severe Covid-19 requiring mechanical ventilation at the intensive care unit, with strongest associations in the younger population. Type 2 diabetes implied a greater risk among women and in those with immigrant background. These findings, originating from high-quality Swedish registries, may be important to direct preventive measures such as vaccination to susceptible patient groups.

For peer review only

For peer review only - http://bmjopen.bmj.com/site/about/guidelines.xhtml

ACKNOWLEDGMENTS

We thank Maria Ioanna Kotopouli, Karolinska Institutet, for expert statistical support.

Funding

This work was supported by grants from the Region Stockholm (ALF-project, grant number 2019-0100).

RH was supported by Region Stockholm (clinical postdoctoral appointment, grant number K 2017-4577) and the Swedish Heart Lung foundation (grant number 20180187)

Authors' contributions

PS had full access to all the data in the study and takes full responsibility for the integrity of the data and accuracy of the data analysis.

Study concept and design: PS, PN, HH.

Acquisition of data: PS

Analysis and interpretation of data: PS, RH, TJ, PN, HH

Drafting the manuscript: PS, RH, PN.

Critical revision of the manuscript for important intellectual content: PS, RH, TJ, PN, HH

Statistical analysis: HH.

Obtained funding: PS

Disclosures

The authors declare that there is no conflict of interest.

For peer review only

Data availability

The data underlying this article cannot be shared publicly due to the privacy of individuals that participated in the study. The data will be shared on reasonable request to the corresponding author.

For peer review only

For peer review only - http://bmjopen.bmj.com/site/about/guidelines.xhtml

REFERENCES

1. Williamson EJ, Walker AJ, Bhaskaran K, et al. Factors associated with COVID-19-related death using OpenSAFELY. *Nature.* 2020;584(7821):430-436.

2. Barron E, Bakhai C, Kar P, et al. Associations of type 1 and type 2 diabetes with COVID-19-related mortality in England: a whole-population study. *The lancet Diabetes & endocrinology.* 2020;8(10):813-822.

3. Stefan N, Birkenfeld AL, Schulze MB, Ludwig DS. Obesity and impaired metabolic health in patients with COVID-19. *Nature reviews Endocrinology.* 2020;16(7):341-342.

4. Zhou F, Yu T, Du R, et al. Clinical course and risk factors for mortality of adult inpatients with COVID-19 in Wuhan, China: a retrospective cohort study. *Lancet (London, England).* 2020.

5. Mancia G, Rea F, Ludergnani M, Apolone G, Corrao G. Renin–Angiotensin–Aldosterone System Blockers and the Risk of Covid-19. *New England Journal of Medicine.* 2020.

6. Fosbol EL, Butt JH, Ostergaard L, et al. Association of Angiotensin-Converting Enzyme Inhibitor or Angiotensin Receptor Blocker Use With COVID-19 Diagnosis and Mortality. *JAMA.* 2020.

7. Petrilli CM, Jones SA, Yang J, et al. Factors associated with hospital admission and critical illness among 5279 people with coronavirus disease 2019 in New York City: prospective cohort study. *BMJ.* 2020;369:m1966.

8. Sattar N, McInnes IB, McMurray JJV. Obesity a Risk Factor for Severe COVID-19 Infection: Multiple Potential Mechanisms. *Circulation.* 2020.

9. Stefan N. Causes, consequences, and treatment of metabolically unhealthy fat distribution. *The Lancet Diabetes & Endocrinology.* 2020;8(7):616-627.

For peer review only - http://bmjopen.bmj.com/site/about/guidelines.xhtml

10. Becker RC. COVID-19 update: Covid-19-associated coagulopathy. *J Thromb Thrombolysis.* 2020;50(1):54-67.

11. Pareek M, Bangash MN, Pareek N, et al. Ethnicity and COVID-19: an urgent public health research priority. *Lancet.* 2020;395(10234):1421-1422.

12. Bornstein SR, Rubino F, Khunti K, et al. Practical recommendations for the management of diabetes in patients with COVID-19. *The Lancet Diabetes & Endocrinology.* 2020;8(6):546-550.

13. Lipsitch M, Swerdlow DL, Finelli L. Defining the Epidemiology of Covid-19 - Studies Needed. *The New England journal of medicine.* 2020;382(13):1194-1196.

14. Holman N, Knighton P, Kar P, et al. Risk factors for COVID-19-related mortality in people with type 1 and type 2 diabetes in England: a population-based cohort study. *The lancet Diabetes & endocrinology.* 2020;8(10):823-833.

15. Ludvigsson JF, Otterblad-Olausson P, Pettersson BU, Ekbom A. The Swedish personal identity number: possibilities and pitfalls in healthcare and medical research. *European Journal of Epidemiology.* 2009;24(11):659-667.

16. Swedish registry for Intensive Care. National Quality Registry for Intensive Care. http://kvalitetsregister.se/englishpages/findaregistry/registerarkivenglish/nationalqualityregistryforintensivecaresir.2175.html. .

17. Ludvigsson JF, Andersson E, Ekbom A, et al. External review and validation of the Swedish national inpatient register. *BMC Public Health.* 2011;11:450.

18. Ludvigsson JF, Svedberg P, Olén O, Bruze G, Neovius M. The longitudinal integrated database for health insurance and labour market studies (LISA) and its use in medical research. *European Journal of Epidemiology.* 2019;34(4):423-437.

For peer review only

19. Oras P, Habel H, Skoglund PH, Svensson P. Elevated Blood Pressure in the Emergency Department: A Risk Factor for Incident Cardiovascular Disease. *Hypertension (Dallas, Tex : 1979).* 2020;75(1):229-236.

20. Wang D, Hu B, Hu C, et al. Clinical Characteristics of 138 Hospitalized Patients With 2019 Novel Coronavirus–Infected Pneumonia in Wuhan, China. *JAMA.* 2020;323(11):1061-1069.

21. Grasselli G, Zangrillo A, Zanella A, et al. Baseline Characteristics and Outcomes of 1591 Patients Infected With SARS-CoV-2 Admitted to ICUs of the Lombardy Region, Italy. *JAMA.* 2020;323(16):1574-1581.

22. Torpy JM, Lynm C, Glass RM. The Metabolic Syndrome. *JAMA.* 2006;295(7):850-850.

23. Schultz WM, Kelli HM, Lisko JC, et al. Socioeconomic Status and Cardiovascular Outcomes. *Circulation.* 2018;137(20):2166-2178.

24. Gao C, Cai Y, Zhang K, et al. Association of hypertension and antihypertensive treatment with COVID-19 mortality: a retrospective observational study. *European Heart Journal.* 2020;41(22):2058-2066.

25. Kreutz R, Algharably EAE, Azizi M, et al. Hypertension, the renin-angiotensin system, and the risk of lower respiratory tract infections and lung injury: implications for COVID-19. *Cardiovasc Res.* 2020.

26. Tziomalos K, Athyros VG, Karagiannis A, Mikhailidis DP. Endothelial dysfunction in metabolic syndrome: prevalence, pathogenesis and management. *Nutrition, metabolism, and cardiovascular diseases : NMCD.* 2010;20(2):140-146.

27. Hotamisligil GS. Inflammation and metabolic disorders. *Nature.* 2006;444(7121):860-867.

For peer review only - http://bmjopen.bmj.com/site/about/guidelines.xhtml

28. Drummond GR, Vinh A, Guzik TJ, Sobey CG. Immune mechanisms of hypertension. *Nature reviews Immunology.* 2019;19(8):517-532.

29. Loperena R, Van Beusecum JP, Itani HA, et al. Hypertension and increased endothelial mechanical stretch promote monocyte differentiation and activation: roles of STAT3, interleukin 6 and hydrogen peroxide. *Cardiovasc Res.* 2018;114(11):1547-1563.

30. Siedlinski M, Jozefczuk E, Xu X, et al. White Blood Cells and Blood Pressure: A Mendelian Randomization Study. *Circulation.* 2020;141(16):1307-1317.

31. Pons S, Fodil S, Azoulay E, Zafrani L. The vascular endothelium: the cornerstone of organ dysfunction in severe SARS-CoV-2 infection. *Critical care (London, England).* 2020;24(1):353.

32. Price-Haywood EG, Burton J, Fort D, Seoane L. Hospitalization and Mortality among Black Patients and White Patients with Covid-19. *New England Journal of Medicine.* 2020;382(26):2534-2543.

33. Lassale C, Gaye B, Hamer M, Gale CR, Batty GD. Ethnic disparities in hospitalisation for COVID-19 in England: The role of socioeconomic factors, mental health, and inflammatory and pro-inflammatory factors in a community-based cohort study. *Brain, behavior, and immunity.* 2020.

34. Rhodes A, Ferdinande P, Flaatten H, Guidet B, Metnitz PG, Moreno RP. The variability of critical care bed numbers in Europe. *Intensive care medicine.* 2012;38(10):1647-1653.

For peer review only - http://bmjopen.bmj.com/site/about/guidelines.xhtml

FIGURE LEGENDS

Figure 1. Associations of cardiometabolic risk factors with severe Covid-19 (adjusted odds ratios with 95% Cis).

†Adjusted for age, sex, educational level, marital status and region of birth.

‡Adjusted for age, sex, educational level, marital status, region of birth and all diagnoses in table 2a

Figure 2. Associations of cardiometabolic risk factors with severe Covid-19 by tertiles of age (adjusted odds ratios with 95% Cis).

Figure 3. Associations of cardiometabolic risk factors with severe Covid-19 by sex (adjusted odds ratios with 95% Cis).

P-values denote likelihood-ratio tests between a model with and one without an interaction term between the indicator variable for the subgroup and the risk factor added to model 2

Figure 4. Associations of cardiometabolic risk factors with severe Covid-19 by region of birth * (adjusted odds ratios with 95% Cis).

*EU 15 comprises of Austria, Belgium, Denmark, Finland, France, Germany, Greece, Ireland, Italy, Luxembourg, Netherlands, Portugal, Spain, Sweden, United Kingdom. The Nordic countries include Denmark, Finland, Iceland, Norway, and Sweden.

P-values denote likelihood-ratio tests between a model with and one without an interaction term between the indicator variable for the subgroup and the risk factor added to model 2

.

For peer review only - http://bmjopen.bmj.com/site/about/guidelines.xhtml

For peer review only

APPENDICES

Appendix A. Online Supplementary data

Supplemental methods – register information. 1

eTable 1 – Hypertension definitions. 2

eTable 2 - Missing Data in the Study Population. 3

eTable 3a-c - Baseline characteristics of the study population by age-group. 4-6

eTable 4 - Baseline characteristics of the study population by sex. 7

eTable 5. Baseline characteristics of the study population by region of birth. 8

eTable 6 - Odds ratios for Covid-19 requiring ICU-admission by cardiometabolic factors and other comorbidities (n=1,417 cases and n=14,170 control subjects) 9

Supplemental Figure S1 – Directed Acyclic Graph. 7

Supplemental Figure S2 - Exclusion flowchart. 8

For peer review only

Table 1a. Baseline characteristics of the study population. Characteristics of patients with COVID-19 requiring mechanical ventilation and control subjects

	**COVID-19 (n = 1,086)**	**Control subjects (n = 10,860)**
Age, median (IQR), y	62.0 (52.0-70.0)	62.0 (52.0-70.0)
**Sex:**		
Male, No. (%)	813 (74.9)	8,130 (74.9)
**Sociodemographics** No. (%)		
Education (years)		
≤9	280 (26.8)	2,144 (20.1)
10-12	466 (44.6)	4,805 (45.1)
≥12	300 (28.7)	3,712 (34.8)
Marital status		
Widow	41 (3.8)	405 (3.7)
Married	632 (58.2)	5,641 (51.9)
Single	218 (20.1)	2,802 (25.8)
Separated	195 (18.0)	2,012 (18.5)
Region of birth		
EU 15* and/or Nordics	596 (55.1)	8,411 (77.5)
**Medical history** No. (%)		
Type 1 diabetes	9 (0.8)	39 (0.4)
Type 2 diabetes	276 (25.4)	1,255 (11.6)
Obesity	99 (9.1)	328 (3.0)
Hypertension	547 (50.4)	4,258 (39.2)
Hyperlipidaemia	292 (33.0)	2,100 (28.6)
Chronic kidney disease	40 (3.7)	146 (1.3)
Cardiovascular disease	105 (9.7)	992 (9.1)
Myocardial infarction	55 (5.1)	559 (5.1)
Ischemic stroke	29 (2.7)	274 (2.5)
Peripheral artery disease	24 (2.2)	249 (2.3)
Heart failure	40 (3.7)	329 (3.0)
Atrial fibrillation	65 (6.0)	589 (5.4)
Deep vein thrombosis	40 (3.7)	208 (1.9)
Pulmonary embolism	13 (1.2)	103 (0.9)
Chronic obstructive pulmonary disease	32 (2.9)	237 (2.2)
Asthma	100 (9.2)	376 (3.5)
Malignancy	158 (14.5)	1,740 (16.0)
Rheumatoid arthritis	17 (1.6)	96 (0.9)
Systemic inflammatory disease	33 (3.0)	129 (1.2)
Inflammatory bowel disease	17 (1.6)	159 (1.5)

*EU 15 comprises of Austria, Belgium, Denmark, Finland, France, Germany, Greece, Ireland, Italy, Luxembourg, Netherlands, Portugal, Spain, Sweden, United Kingdom. The Nordic countries include Denmark, Finland, Iceland, Norway, and Sweden.

For peer review only

Table 1b. Pharmacological treatments of the study population.

	Covid-19 (n = 1,086)	Control subjects (n = 10,860)
**Treatments** No. (%)		
Antihypertensive treatments		
ACE inhibitors	168 (15.5)	1,310 (12.1)
ARBs	218 (20.1)	1,694 (15.6)
Calcium-channel blockers	239 (22.0)	1,648 (15.2)
Beta-blockers	222 (20.4)	1,849 (17.0)
Diuretics	51 (4.7)	522 (4.8)
Antidiabetic treatments		
Any antidiabetics	240 (22.1)	1,144 (10.5)
Insulins	80 (7.4)	391 (3.6)
Biguanides	200 (18.4)	855 (7.9)
Sulfonylureas	28 (2.6)	93 (0.9)
Glitazons	6 (0.6)	10 (0.1)
DPP-4 inhibitors	44 (4.1)	210 (1.9)
GLP-1 RAs	37 (3.4)	184 (1.7)
SGLT-2 inhibitors	46 (4.2)	184 (1.7)
Meglitinides	4 (0.4)	34 (0.3)
Statins	288 (26.5)	2,242 (20.6)
Aspirin	136 (12.5)	1,103 (10.2)
Other Antiplatelet drugs	20 (1.8)	234 (2.2)
Warfarin	17 (1.6)	150 (1.4)
NOAC	45 (4.1)	474 (4.4)

Abbreviations: ACE-inhibitors, angiotensin converting enzyme-inhibitors; ARBs, angiotensin receptor blockers; DPP-4 inhibitors, dipeptidyl peptidase-4 inhibitors; GLP-1 RAs, glucagon-like peptide-1 receptor agonists; SGLT2 inhibitors, sodium-glucose cotransporter-2 inhibitors NOAC, new oral anticoagulants

For peer review only

Table 2a. Odds ratios for Covid-19 requiring mechanical ventilation by cardiometabolic factors and other comorbidities.

	Adjusted for age and sex			Adjusted model 2†			Adjusted model 3 ‡		
Risk factors	OR	95% CI	p-value	OR	95% CI	p-value	OR	95% CI	p-value
Type 1 diabetes	2.32	1.12-4.81	0.023	3.13	1.52-6.42	0.002	2.56	1.25-5.24	0.010
Type 2 diabetes	2.73	2.33-3.20	<0.001	2.25	1.90-2.65	<0.001	1.81	1. 49-2.19	<0.001
Obesity	3.23	2.56-4.08	<0.001	3.13	2.43-4.02	<0.001	2.03	1.55-2.65	<0.001
Hypertension	1.76	1.52-2.05	<0.001	1.73	1.48-2.01	<0.001	1.26	1.05--1.51	<0.013
Hyperlipidaemia	1.35	1.15-1.58	<0.001	1.22	1.03-1.43	<0.018	0.90	0.75-1.09	0.286
CKD	2.83	1.97-4.05	<0.001	2.51	1.69-3.70	<0.001	1.84	1.21-2.82	0.005
CVD	1.07	0.86-1.33	0.554	1.03	0.82-1.29	0.789	0.75	0.58-0.96	0.022
Heart failure	1.23	0.87-1.73	0.236	1.13	0.79-1.62	0.48	0.78	0.51-1.19	0.253
Atrial fibrillation	1.11	0.85-1.46	0.43	1.24	0.93-1.64	0.136	1.04	0.75-1.43	0.819
VTE	1.74	1.27-2.39	0.001	1.90	1.37-2.62	<0.001	1.65	1.18-2.31	0.004
COPD	1.37	0.94-1.99	0.105	1.34	0.91-1.97	0.133	0.87	0.56-1.36	0.552
Asthma	2.84	2.26-3.58	<0.001	2.78	2.18-3.53	<0.001	2.25	1.74-2.90	<0.001
Malignancy	0.89	0.74-1.06	0.195	0.97	0.80-1.17	0.725	0.85	0.70-1.04	0.103
Rheumatoid arthritis	1.79	1.06-3.00	0.029	1.86	1.11-3.12	0.019	1.27	0.72-2.23	0.407
Systemic infl. disease	2.65	1.79-3.92	<0.001	2.57	1.71-3.86	<0.001	1.96	1.28-2.99	0.002
Infl. bowel disease	1.07	0.65-1.77	0.792	1.21	0.72-2.03	0.479	0.94	0.54-1.64	0.839

†Adjusted for age, sex, educational level, marital status and region of birth.
‡Adjusted for age, sex, educational level, marital status and region of birth and all diagnoses in table 2a

For peer review only - http://bmjopen.bmj.com/site/about/guidelines.xhtml

Abbreviations CVD, cardiovascular disease; VTE, venous thromboembolism; COPD, chronic obstructive pulmonary disease; CKD, chronic kidney disease.

For peer review only

For peer review only - http://bmjopen.bmj.com/site/about/guidelines.xhtml

Table 2b. Odds ratios for Covid-19 requiring mechanical ventilation by pharmacological treatments.

	Adjusted for age and sex			Adjusted model 2†			Adjusted model 3 ‡		
**Treatments**	OR	95% CI	p-value	OR	95% CI	p-value	OR	95% CI	p-value
Antihypertensive treatments									
ACE-inhibitors	1.35	1.13-1.62	0.001	1.35	1.12-1.62	0.797	0.99	0.81-1.22	0.931
ARBs	1.39	1.18-1.64	<0.001	1.47	1.24-1.75	<0.001	1.07	0.88-1.30	0.474
CCBs	1.64	1.39-1.93	<0.001	1.64	1.39-1.93	<0.001	1.25	1.03-1.52	0.025
Beta-blockers	1.28	1.08-1.51	0.004	1.27	1.07-1.51	0.007	0.90	0.73-1.11	0.345
Diuretics	0.98	0.72-1.32	0.870	1.00	0.74-1.36	0.996	0.74	0.53-1.03	0.078
Antidiabetic treatments									
Insulins	2.15	1.67-2.77	<0.001	1.90	1.46-2.47	<0.001	0.85	0.62-1.16	0.305
Biguanides	2.72	2.28-3.24	<0.001	2.26	1.88-2.72	<0.001	1.40	1.01-1.94	0.044
Sulfonylureas	3.08	2.01-4.73	<0.001	2.13	1.33-3.41	0.002	1.17	0.72-1.91	0.530
Glitazons	6.03	2.19-16.6	0.001	5.46	1.98-15.0	0.001	2.86	1.04-7.85	0.042
DPP-4 inhibitors	2.16	1.54-3.01	<0.001	1.77	1.25-2.50	0.001	0.91	0.62-1.33	0.632
GLP-1 RA	2.79	1.92-4.03	<0.001	2.71	1.85-3.97	<0.001	1.24	0.82-1.87	0.312
SGLT-2 inhibitors	2.58	1.85-3.59	<0.001	2.30	1.63-3.25	<0.001	1.20	0.82-1.74	0.346
Meglitinides	1.18	0.42-3.33	0.383	1.03	0.36-2.96	0.953	0.55	0.19-1.61	0.276
Statins	1.44	1.23-1.69	<0.001	1.32	1.12-1.55	0.001	0.84	0.63-1.13	0.247
Aspirin	1.29	1.05-1.57	0.013	1.14	0.93-1.41	0.200	0.97	0.75-1.24	0.791
Warfarin	1.14	0.68-1.90	0.622	1.29	0.76-2.17	0.347	0.96	0.52-1.76	0.894
NOAC	0.95	0.69-1.30	0.730	1.04	0.75-1.44	0.806	0.70	0.46-1.06	0.093

For peer review only

For peer review only - http://bmjopen.bmj.com/site/about/guidelines.xhtml

†Adjusted for age, sex, educational level, marital status and region of birth.
‡Adjusted for age, sex, educational level, marital status and region of birth and all diagnoses in table 2a
Abbreviations: ACE-inhibitors, angiotensin converting enzyme-inhibitors; ARBs, angiotensin receptor blockers; CCBs, calcium-channel blockers; DPP-4 inhibitors, dipeptidyl peptidase-4 inhibitors; GLP-1 RA, glucagon-like peptide-1 receptor agonists; SGLT2 inhibitors, sodium-glucose cotransporter-2 inhibitors; NOAC, new oral anticoagulants

For peer review only

For peer review only - http://bmjopen.bmj.com/site/about/guidelines.xhtml

Table 3. Covid-19 risk factors by subgroups of age, sex and region of birth. Odds ratios for Covid-19 receiving mechanical ventilation compared to matched control subjects by tertiles of age, sex and region of birth (EU15).

	Type 2 diabetes	Obesity	Hypertension	CKD	CVD	VTE	Asthma	SID	Heart Failure
**Age**									
21-56 years	4.5 (3.3-6.2)	7.6 (5.3-11.0)	2.6 (2.0-3.3)	7.7 (3.4-17.5)	1.6 (0.8-3.3)	3.9 (2.0-7.6)	4.9 (3.3-7.3)	6.7 (3.0-14.9)	5.4 (2.4-12.2)
57-67 years	2.6 (2.0-3.4)*	3.7 (2.5-5.4)*	1.7 (1.3-2.1)*	3.2 (1.6-6.4)	1.4 (1.0-2.0)	1.9 (1.1-3.2)	3.7 (2.7-5.1)	2.5 (1.1-5.5)	1.5 (0.8-2.6)*
68-87 years	1.4 (1.1-1.9)*	2.2 (1.3-3.7)*	1.3 (1.0-1.7)*	1.5 (0.8-2.7)*	0.9 (0.7-1.2)	1.4 (0.8-2.3)*	2.3 (1.5-3.5)*	2.0 (1.2-3.7)*	0.8 (0.5-1.4)*
**Sex**									
Male	2.0 (1.7-2.4)	3.1 (2.3-4.3)	1.7 (1.4-2.0)	2.2 (1.4-3.4)	1.0 (0.8-1.3)	1.8 (1.3-2.6)	2.3 (1.7-3.1)	2.1 (1.1-3.9)	1.0 (0.7-1.5)
female	3.5 (2.5-5.0)	3.2 (2.0-5.0)	1.8 (1.3-2.5)	4.2 (1.9-9.3)	1.2 (0.7-2.2)	2.2 (1.1-4.2)	3.9 (2.6-5.8)	3.1 (1.8-5.3)	2.0 (0.9-4.4)
p-value	0.001	0.922	0.327	0.113	0.416	0.558	0.030	0.019	0.103
**Region of birth**									
Outside EU15	3.3 (2.6-4.2)	2.3 (1.6-3.4)	1.8 (1.5-2.3)	2.5 (1.4-4.6)	1.4 (1.0-1.9)	1.4 (0.8-2.4)	3.2 (2.2-4.5)	2.1 (1.1-4.0)	1.3 (0.8-2.31)
EU 15/Nordic	2.0 (1.6-2.6)	3.7 (2.7-5.1)	1.6 (1.3-1.9)	2.6 (1.6-4.2)	0.9 (0.7-1.2)	2.0 (1.4-3.0)	2.7 (1.9-3.7)	2.8 (1.7-4.6)	1.1 (0.7-1.7)
p-value	0.004	0.066	0.476	0.872	0.072	0.248	0.584	0.584	0.651

P-values denote likelihood-ratio tests between a model with and one without an interaction term between the indicator variable for the subgroup and the risk factor added to model 2. Age subgroups were compared to 21-56 years, p-values for interaction are presented as * p-value<0.05. **Abbreviations:** CKD, chronic kidney disease, CVD, cardiovascular disease (history of myocardial infarction, ischemic stroke or peripheral arterial disease); VTE, venous thromboembolic disease. SID, Systemic inflammatory disease.

For peer review only - http://bmjopen.bmj.com/site/about/guidelines.xhtml

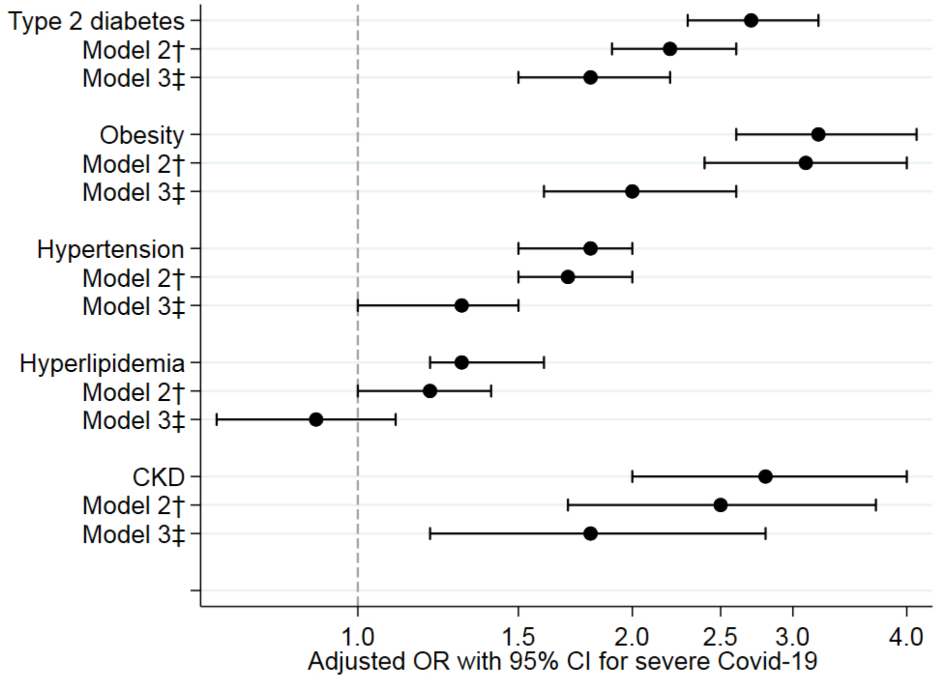

Associations of cardiometabolic risk factors with severe Covid-19 (adjusted odds ratios with 95% Cis).

364x265mm (72 x 72 DPI)

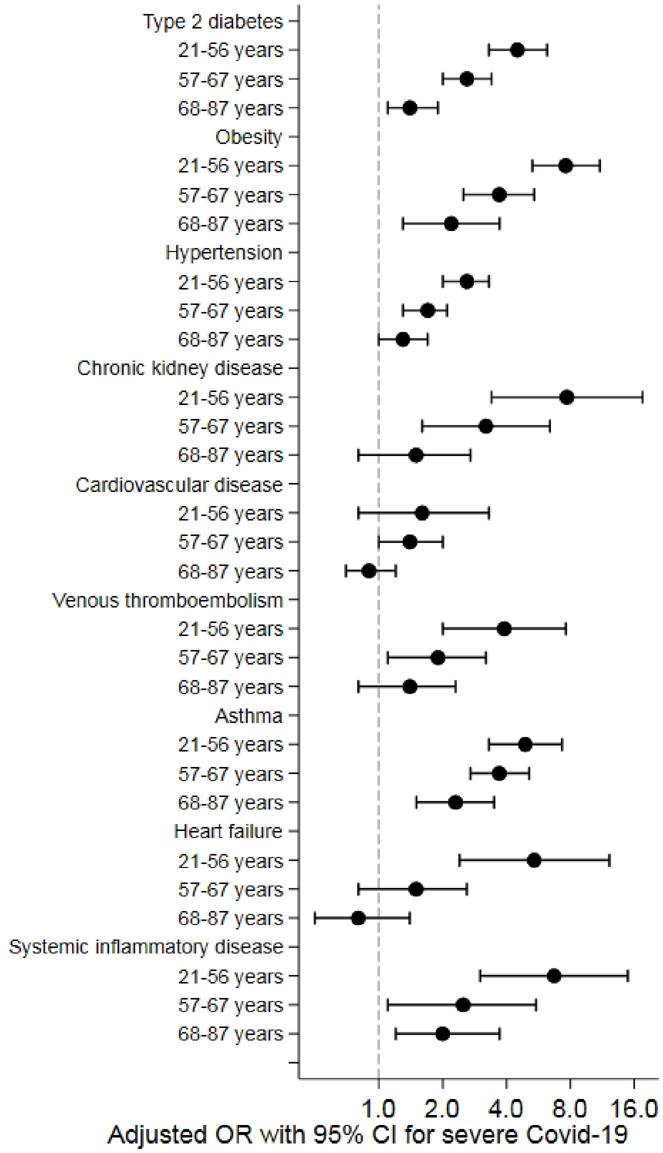

Figure 2. Associations of cardiometabolic risk factors with severe Covid-19 by age tertiles (adjusted odds ratios with 95% Cis).

181x297mm (72 x 72 DPI)

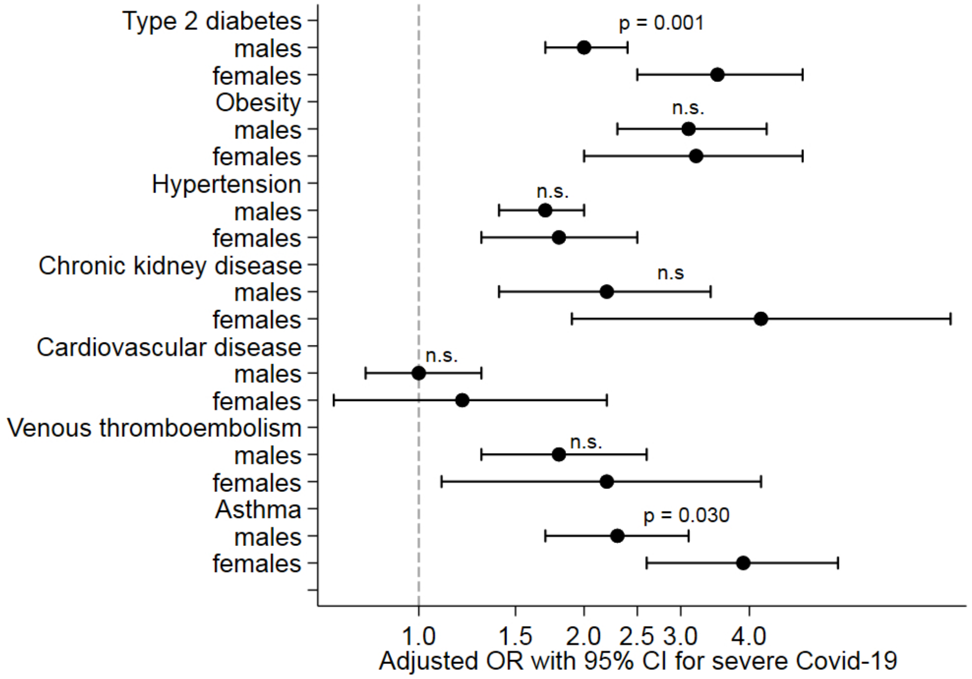

Associations of cardiometabolic risk factors with severe Covid-19 by sex (adjusted odds ratios with 95% Cis).

358x260mm (72 x 72 DPI)

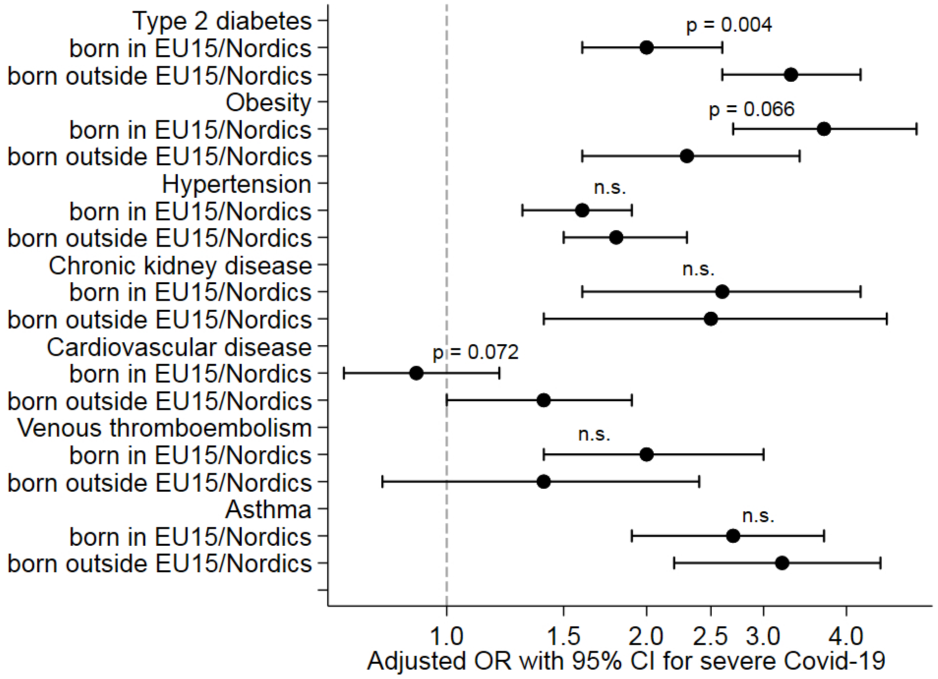

Associations of cardiometabolic risk factors with severe Covid-19 by region of birth * (adjusted odds ratios with 95% Cis).

358x260mm (72 x 72 DPI)

Online Supplementary data

Cardiometabolic factors and risk for severe COVID-19 requiring invasive mechanical ventilation during the Swedish epidemic

Svensson P, Hofmann R, Habel H, Jernberg T, Nordberg P

For peer review only

Table of contents

Supplemental methods – register information 1

eTable 1 – Hypertension definitions 2

eTable 2 - Missing Data in the Study Population... 3

eTable 3a-c - Baseline characteristics of the study population by age-group……………………………………4-6

eTable 4 - Baseline characteristics of the study population by sex 7

eTable 5 - Baseline characteristics of the study population by region of birth...…………………………………..8

eTable 6 - Odds ratios for Covid-19 requiring ICU-admission by cardiometabolic factors and other comorbidities (n=1,417 cases and n=14,170 control subjects)..…………………………………………………………...……..9

Supplemental figure S1 - Directed Acyclic Graph……………………………………………………………………..10

Supplemental figure S2 - Exclusion flowchart ……………………………………………………………………..11

References………………………………………………………………………………………………………..12

Supplemental methods – register information

The Population Register

The Population Register is managed by Statistics Sweden and includes information on deaths, emigration and immigration for the entire Swedish population. All residents are assigned a unique personal identity number that can be used for linkage of different data resources including several national health registers of high quality.

The longitudinal integrated database for health insurance and labor market studies (LISA)

LISA is managed by Statistics Sweden and includes annual measurements on several socioeconomic and sociodemographic variables, including income, education and country of birth.

The Swedish Patient Register

Swedish Patient Register is managed by the National Board of Health and Welfare and covers inpatient care since 1964- (nationwide since 1987) and non-primary outpatient care since 2001. The register is nationwide with a near complete coverage during the study period.

The register is regulated by the Health Care Data Register Act (1998:543; Lag om hälsodataregister) and the Patient Register ordinance (2001:707; Förordning om patientregister hos Socialstyrelsen). It is mandatory for all physicians, private and publicly funded, to deliver data to the Patient Register. Data from the Patient Register are subjugated to the Health and Medical Services Act (1982:763; Hälso och sjukvårdslag) and the Patient Data Act (2008:355; Patientdatalag). [1]

The Swedish Prescribed Drug Register

The Swedish Prescribed Drug Register is managed by the National Board of Health and Welfare and started on July 1, 2005. The register covers all drugs except over-the-counter medication (which is not covered at all) and medications administered at hospitals (which is only covered to some extent in the Prescribed Drug Register and completely covered through the National Patient Register in some counties).

National Board of Health and Welfare register data is protected by strict confidentiality but can be made available for research after a special review after which data can be provided to Swedish researchers according to standard legal provisions and procedures. Of special importance to the regulation of Swedish medical research and health care is also the Public Access to Information and Secrecy Act (2009:400, Offentlighets- och sekretesslagen). [1]

For peer review only

eTable 1 – Hypertension definition.

**Anti-hypertensive drugs**	**ATC-codes**	**Exclusion diagnosis (ICD-10)**
Diuretics	C03A, C03D, C03E	
Beta-blockers *	C07A, C07F	Angina pectoris (I208, I209)<br>Atrial fibrillation (I48)<br>MI (I21, I22)<br>Heart failure (I50)
Calcium channel blockers	C08C, C08D	
ACE-inhibitors †	C09A, C09B	Heart failure (I50)
Angiotensin receptor blockers ‡	C09C, C09DA, C09DB	Heart failure (I50)
Other drugs targeting blood vessels	C02C, C02D	

Patients with a pick-up of a prescription of the anti-hypertensive drugs within the preceding 12 months of the index date were considered as having hypertension. Beta-blockers, ACE-inhibitors, and Angiotensin receptor blockers may be prescribed for other diagnoses than hypertension. Patients were not classified as having hypertension if these drugs were found in combinations with any such diagnosis. An existing record of hypertension (I109) was superior to the pick-ups of prescribed drugs.

* Patients with a diagnosis of angina pectoris (I208, I209), atrial fibrillation (I48), MI (I21, I22) or heart failure (I50) and simultaneously prescribed with beta-blockers were not classified as having hypertension.

† ‡ Patients with a diagnosis of heart failure (I50) with concurrently prescription of ACE inhibitors or angiotensin receptor blockers were not classified as having hypertension.

For peer review only

eTable 2 - Missing Data[*] in the Study Cohort

Characteristic	Missing data, No. (%)
Age	0 (0.0)
Sex	0 (0.0)
Level of education	239 (2.0)
Region of birth	5 (0.1)
Fills of prescriptions	0 (0)
Medical history	0 (0)

For peer review only

eTable 3a. Baseline characteristics of the study population in the youngest tertile. Characteristics of patients with Covid-19 requiring mechanical ventilation and control subjects

Youngest tertile (age 21-56 years)

	**Covid-19 (n = 367)**	**Control subjects (n = 3,670)**
Age, median (IQR), y	49 (42-53)	49 (42-53)
**Sex:**		
Male, No. (%)	261 (71.1)	2,610 (71.1)
**Sociodemographics** No. (%)		
Education (years)		
≤9	78 (22.5)	529 (14.8)
10-12	175 (50.4)	1,633 (45.7)
≥12	94 (27.1)	1,409 (39.5)
Marital status		
Unmarried	169 (46.0)	1,911 (52.1)
Married	198 (54.0)	1,759 (47.9)
Region of birth		
EU 15* and/or Nordics	173 (47.3)	2,498 (68.1)
**Medical history** No. (%)		
Type 1 diabetes	3 (0.8)	22 (0.6)
Type 2 diabetes	78 (21.3)	161 (4.4)
Obesity	64 (17.4)	105 (2.9)
Hypertension	102 (27.8)	473 (12.9)
Hyperlipidaemia	53 (14.4)	230 (6.3)
Chronic kidney disease	13 (3.5)	14 (0.4)
Cardiovascular disease	9 (2.5)	53 (1.4)
Myocardial infarction	7 (1.9)	35 (1.0)
Ischemic stroke	2 (0.5)	15 (0.4)
Peripheral artery disease	1 (0.3)	13 (0.4)
Heart failure	9 (2.5)	17 (0.5)
Atrial fibrillation	8 (2.2)	34 (0.9)
Deep vein thrombosis	12 (3.3)	31 (0.8)
Pulmonary embolism	2 (0.5)	9 (0.2)
Chronic obstructive pulmonary disease	2 (0.5)	14 (0.4)
Asthma	41 (11.2)	100 (2.7)
Malignancy	21 (5.7)	240 (6.5)
Rheumatoid arthritis	4 (1.1)	22 (0.6)
Systemic inflammatory disease	8 (2.2)	14 (0.4)
Inflammatory bowel disease	3 (0.8)	55 (1.5)

* EU 15 comprises of Austria, Belgium, Denmark, Finland, France, Germany, Greece, Ireland, Italy, Luxembourg, Netherlands, Portugal, Spain, Sweden, United Kingdom. The Nordic countries include Denmark, Finland, Iceland, Norway, and Sweden.

For peer review only

For peer review only - http://bmjopen.bmj.com/site/about/guidelines.xhtml

eTable 3b. Baseline characteristics of the study population in the mid age-group tertile. Characteristics of patients with Covid-19 requiring mechanical ventilation and control subjects

Mid tertile (age 57-67 years)

	**Covid-19 (n = 379)**	**Control subjects (n = 3,790)**
Age, median (IQR), y	62 (59-65)	62 (59-65)
**Sex:**		
Male, No. (%)	282 (74.4)	2,820 (74.4)
**Sociodemographics** No. (%)		
Education (years)		
≤9	94 (25.1)	697 (18.6)
10-12	163 (43.5)	1,745 (46.5)
≥12	118 (31.5)	1,310 (34.9)
Marital status		
Unmarried	151 (39.8)	1,856 (49.0)
Married	228 (60.2)	1,934 (51.0)
Region of birth		
EU 15* and/or Nordics	195 (51.6)	2,914 (76.9)
**Medical history** No. (%)		
Type 1 diabetes	5 (1.3)	10 (0.3)
Type 2 diabetes	113 (29.8)	463 (12.2)
Obesity	12 (3.2)	75 (2.0)
Hypertension	207 (54.6)	1,578 (41.6)
Hyperlipidaemia	114 (30.1)	770 (20.3)
Chronic kidney disease	14 (3.7)	46 (1.2)
Cardiovascular disease	44 (11.6)	311 (8.2)
Myocardial infarction	26 (6.9)	185 (4.9)
Ischemic stroke	11 (2.9)	81 (2.1)
Peripheral artery disease	8 (2.1)	78 (2.1)
Heart failure	15 (4.0)	96 (2.5)
Atrial fibrillation	19 (5.0)	140 (3.7)
Deep vein thrombosis	13 (3.4)	83 (2.2)
Pulmonary embolism	5 (1.3)	40 (1.1)
Chronic obstructive pulmonary disease	12 (3.2)	75 (2.0)
Asthma	42 (11.1)	138 (3.6)
Malignancy	53 (14.0)	538 (14.2)
Rheumatoid arthritis	9 (2.4)	30 (0.8)
Systemic inflammatory disease	11 (2.9)	39 (1.0)
Inflammatory bowel disease	5 (1.3)	52 (1.4)

* EU 15 comprises of Austria, Belgium, Denmark, Finland, France, Germany, Greece, Ireland, Italy, Luxembourg, Netherlands, Portugal, Spain, Sweden, United Kingdom. The Nordic countries include Denmark, Finland, Iceland, Norway, and Sweden.

For peer review only

eTable 3c. Baseline characteristics of the study population in the oldest tertile. Characteristics of patients with Covid-19 requiring mechanical ventilation and control subjects

Oldest tertile (age 68-87 years)

	**Covid-19 (n = 340)**	**Control subjects (n = 3,400)**
Age, median (IQR), y	73 (70-76)	73 (70-76)
**Sex:**		
Male, No. (%)	270 (79.4)	2,700 (79.4)
**Sociodemographics** No. (%)		
Education (years)		
≤9	108 (33.3)	918 (27.5)
10-12	128 (39.5)	1,427 (42.8)
≥12	88 (27.2)	993 (29.7)
Marital status		
Unmarried	134 (39.4)	1,452 (42.7)
Married	206 (60.6)	1,948 (57.3)
Region of birth		
EU 15* and/or Nordics	228 (67.5)	2,999 (88.2)
**Medical history** No. (%)		
Type 1 diabetes	1 (0.3)	7 (0.2)
Type 2 diabetes	96 (28.2)	631 (18.6)
Obesity	20 (5.9)	97 (2.9)
Hypertension	243 (71.5)	2,207 (64.9)
Hyperlipidaemia	124 (36.5)	1,101 (32.4)
Chronic kidney disease	14 (4.1)	86 (2.5)
Cardiovascular disease	60 (17.6)	630 (18.5)
Myocardial infarction	22 (6.5)	341 (10.0)
Ischemic stroke	20 (5.9)	179 (5.3)
Peripheral artery disease	16 (4.7)	159 (4.7)
Heart failure	20 (5.9)	216 (6.4)
Atrial fibrillation	57 (16.8)	415 (12.2)
Deep vein thrombosis	15 (4.4)	94 (2.8)
Pulmonary embolism	6 (1.8)	54 (1.6)
Chronic obstructive pulmonary disease	22 (6.5)	149 (4.4)
Asthma	33 (9.7)	139 (4.1)
Malignancy	15 (4.4)	94 (2.8)
Rheumatoid arthritis	4 (1.2)	44 (1.3)
Systemic inflammatory disease	15 (4.4)	76 (2.2)
Inflammatory bowel disease	10 (2.9)	52 (1.5)

* EU 15 comprises of Austria, Belgium, Denmark, Finland, France, Germany, Greece, Ireland, Italy, Luxembourg, Netherlands, Portugal, Spain, Sweden, United Kingdom. The Nordic countries include Denmark, Finland, Iceland, Norway, and Sweden.

For peer review only

For peer review only - http://bmjopen.bmj.com/site/about/guidelines.xhtml

eTable 4. Baseline characteristics of the study population by sex. Characteristics of patients with Covid-19 requiring mechanical ventilation and control subjects

	Males		Females	
	**Covid-19 (n = 813)**	**Control subjects (n = 8,130)**	**Covid-19 (n = 273)**	**Control subjects (n = 2,730)**
Age, median (IQR), y	62 (54-70)	62 (54-70)	60 (50-68)	60 (50-68)
**Sociodemographics** No. (%)				
Education (years)				
≤9	204 (25.9)	1,678 (21.0)	76 (29.5)	466 (17.3)
10-12	351 (44.5)	3,606 (45.2)	115 (44.6)	1,199 (44.6)
≥12	233 (29.6)	2,691 (33.7)	67 (26.0)	1,021 (38.0)
Marital status				
Unmarried	314 (38.6)	3,760 (46.2)	140 (51.3)	1,459 (53.4)
Married	499 (61.4)	4,370 (53.8)	133 (48.7)	1,271 (46.6)
Region of birth				
EU 15* and/or Nordics	439 (54.3)	6,250 (76.9)	157 (57.5)	2,161 (79.2)
**Medical history** No. (%)				
Diabetes mellitus	216 (26.6)	1,099 (13.5)	69 (25.3)	195 (7.1)
Obesity	63 (7.7)	218 (2.7)	36 (13.2)	110 (4.0)
Hypertension	419 (51.5)	3,332 (41.0)	128 (46.9)	926 (33.9)
Hyperlipidaemia	103 (12.7)	667 (8.2)	26 (9.5)	131 (4.8)
Chronic kidney disease	26 (3.2)	121 (1.5)	14 (5.1)	25 (0.9)
Cardiovascular disease	91 (11.2)	877 (10.8)	14 (5.1)	115 (4.2)
Myocardial infarction	51 (6.3)	521 (6.4)	4 (1.5)	38 (1.4)
Ischemic stroke	22 (2.7)	225 (2.8)	7 (2.6)	49 (1.8)
Peripheral artery disease	19 (2.3)	216 (2.7)	5 (1.8)	33 (1.2)
Heart failure	30 (3.7)	286 (3.5)	10 (3.7)	43 (1.6)
Atrial fibrillation	53 (6.5)	510 (6.3)	12 (4.4)	79 (2.9)
Deep vein thrombosis	31 (3.8)	163 (2.0)	9 (3.3)	45 (1.6)
Pulmonary embolism	9 (1.1)	81 (1.0)	4 (1.5)	22 (0.8)
Chronic obstructive pulmonary disease	24 (3.0)	184 (2.3)	8 (2.9)	53 (1.9)
Asthma	58 (7.1)	256 (3.1)	42 (15.4)	120 (4.4)
Malignancy	122 (15.0)	1,287 (15.8)	36 (13.2)	453 (16.6)
Rheumatoid arthritis	9 (1.1)	54 (0.7)	8 (2.9)	42 (1.5)
Systemic inflammatory disease	13 (1.6)	64 (0.8)	20 (7.3)	65 (2.4)
Inflammatory bowel disease	12 (1.5)	109 (1.3)	5 (1.8)	50 (1.8)

* EU 15 comprises of Austria, Belgium, Denmark, Finland, France, Germany, Greece, Ireland, Italy, Luxembourg, Netherlands, Portugal, Spain, Sweden, United Kingdom. The Nordic countries include Denmark, Finland, Iceland, Norway, and Sweden.

For peer review only

eTable 5. Baseline characteristics of the study population by region of birth. Characteristics of patients with Covid-19 requiring mechanical ventilation and control subjects

	**Region of birth within EU15/ and/or Nordic Countries**		**Region of birth outside EU15/ and/or Nordic Countries**	
	**Covid-19 (n = 596)**	**Control subjects (n = 5,960)**	**Covid-19 (n = 490)**	**Control subjects (n = 4,900)**
Age, median (IQR), y	64 (54-72)	64 (54-72)	59 (51-67)	59 (51-67)
**Sex:**				
Male, No. (%)	439 (73.7)	4,390 (73.7)	374 (76.3)	3,740 (76.3)
**Sociodemographics** No. (%)				
Education (years)				
≤9	121 (20.6)	1,161 (19.8)	159 (34.6)	983 (20.5)
10-12	295 (50.3)	2,636 (44.9)	171 (37.3)	2,169 (45.2)
≥12	171 (29.1)	2,069 (35.3)	129 (28.1)	1,643 (34.3)
Marital status				
Unmarried	299 (50.2)	2,859 (48.0)	155 (31.6)	2,360 (48.2)
Married	297 (49.8)	3,101 (52.0)	335 (68.4)	2,540 (51.8)
**Medical history** No. (%)				
Diabetes mellitus	128 (21.5)	699 (11.7)	157 (32.0)	595 (12.1)
Obesity	60 (10.1)	170 (2.9)	39 (8.0)	158 (3.2)
Hypertension	313 (52.5)	2,547 (42.7)	234 (47.8)	1,711 (34.9)
Hyperlipidaemia	75 (12.6)	496 (8.3)	54 (11.0)	302 (6.2)
Chronic kidney disease	21 (3.5)	83 (1.4)	19 (3.9)	63 (1.3)
Cardiovascular disease	57 (9.6)	622 (10.4)	48 (9.8)	370 (7.6)
Myocardial infarction	29 (4.9)	338 (5.7)	26 (5.3)	221 (4.5)
Ischemic stroke	16 (2.7)	174 (2.9)	13 (2.7)	100 (2.0)
Peripheral artery disease	15 (2.5)	158 (2.7)	9 (1.8)	91 (1.9)
Heart failure	21 (3.5)	192 (3.2)	19 (3.9)	137 (2.8)
Atrial fibrillation	53 (8.9)	373 (6.3)	12 (2.4)	216 (4.4)
Deep vein thrombosis	25 (4.2)	112 (1.9)	15 (3.1)	96 (2.0)
Pulmonary embolism	8 (1.3)	64 (1.1)	5 (1.0)	39 (0.8)
Chronic obstructive pulmonary disease	18 (3.0)	125 (2.1)	14 (2.9)	112 (2.3)
Asthma	53 (8.9)	212 (3.6)	47 (9.6)	164 (3.3)
Malignancy	105 (17.6)	1,075 (18.0)	53 (10.8)	665 (13.6)
Rheumatoid arthritis	9 (1.5)	64 (1.1)	8 (1.6)	32 (0.7)
Systemic inflammatory disease	21 (3.5)	77 (1.3)	12 (2.4)	52 (1.1)
Inflammatory bowel disease	14 (2.3)	89 (1.5)	3 (0.6)	70 (1.4)

* EU 15 comprises of Austria, Belgium, Denmark, Finland, France, Germany, Greece, Ireland, Italy, Luxembourg, Netherlands, Portugal, Spain, Sweden, United Kingdom. The Nordic countries include Denmark, Finland, Iceland, Norway, and Sweden.

For peer review only - http://bmjopen.bmj.com/site/about/guidelines.xhtml

eTable 6. Odds ratios for Covid-19 requiring ICU-admission by cardiometabolic factors and other comorbidities (n=1,417 cases and n=14,170 control subjects).

	Adjusted for age and sex			Adjusted model 2†			Adjusted model 3‡		
Risk factors	OR	95% CI	p-value	OR	95% CI	p-value	OR	95% CI	p-value
Type 1 diabetes	2.18	1.19-3.98	0.011	2.84	1.55-5.21	0.001	2.83	1.51-5.30	0.001
Type 2 diabetes	2.88	2.51-3.31	<0.001	2.42	2.09-2.79	<0.001	1.94	1.64-2.31	<0.001
Obesity	4.17	3.46-5.02	<0.001	4.12	3.39-5.02	<0.001	2.91	2.34-3.60	<0.001
Hypertension	1.83	1.61-2.09	<0.001	1.82	1.59-2.08	<0.001	1.41	1.21-1.65	<0.001
Hyperlipidaemia	1.56	1.36-1.79	<0.001	1.47	1.28-1.70	<0.001	0.87	0.74-1.03	0.104
CKD	3.22	2.39-4.33	<0.001	2.91	2.12-3.99	<0.001	1.98	1.36-2.86	<0.001
CVD	1.18	0.98-1.42	0.089	1.12	0.93-1.36	0.240	0.72	0.58-0.90	0.004
Heart failure	1.62	1.24-2.12	<0.001	1.53	1.15-2.03	0.003	0.87	0.62-1.24	0.442
Atrial fibrillation	1.53	1.24-1.90	<0.001	1.69	1.36-2.10	0.001	1.32	1.02-1.71	0.035
VTE	1.49	1.12-1.99	0.006	1.62	1.21-2.17	<0.001	1.39	1.02-1.90	0.039
COPD	1.89	1.42-2.52	<0.001	1.85	1.37-2.49	<0.001	1.07	0.74-1.54-	0.720
Asthma	3.33	2.76-4.02	<0.001	3.39	2.79-4.13	<0.001	2.91	2.35-3.62	<0.001
Malignancy	0.99	0.85-1.16	0.938	1.08	0.92-1.26	0.358	0.98	0.83-1.16	0.837
Rheumatoid arthritis	2.08	1.35-3.18	0.001	2.22	1.45-3.39	<0.001	1.68	1.03-2.74	0.038
Systemic infl. disease	2.60	1.85-3.64	<0.001	2.51	1.77-3.57	<0.001	1.85	1.27-2.70	0.001
Infl. bowel disease	0.98	0.62-1.53	0.917	1.10	0.69-1.75	0.688	1.00	0.60-1.64	0.986

†Adjusted for age, sex, educational level, marital status and region of birth.
‡Adjusted for age, sex, educational level, marital status and region of birth and all diagnoses in table 2a.

For peer review only - http://bmjopen.bmj.com/site/about/guidelines.xhtml

Supplemental Fig. S1 – Directed Acyclic Graph

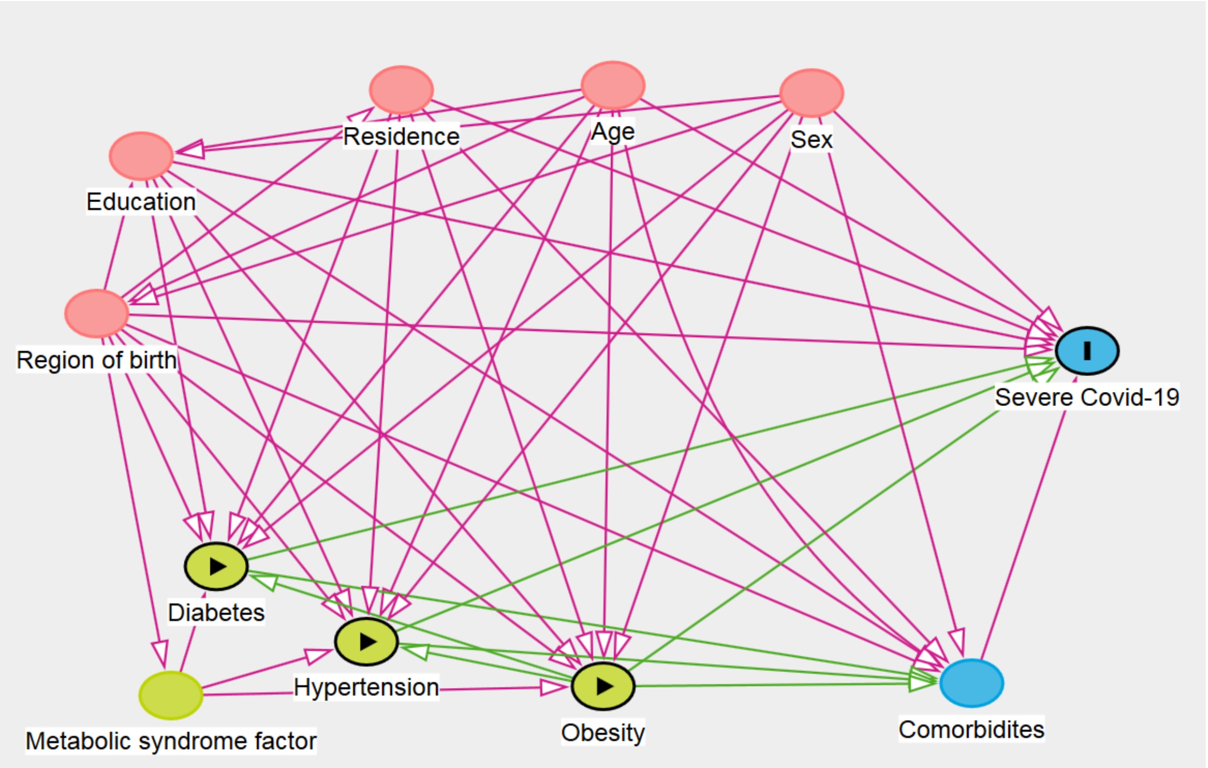

review only

Supplemental Fig. S2 - Flow chart of exclusion criteria

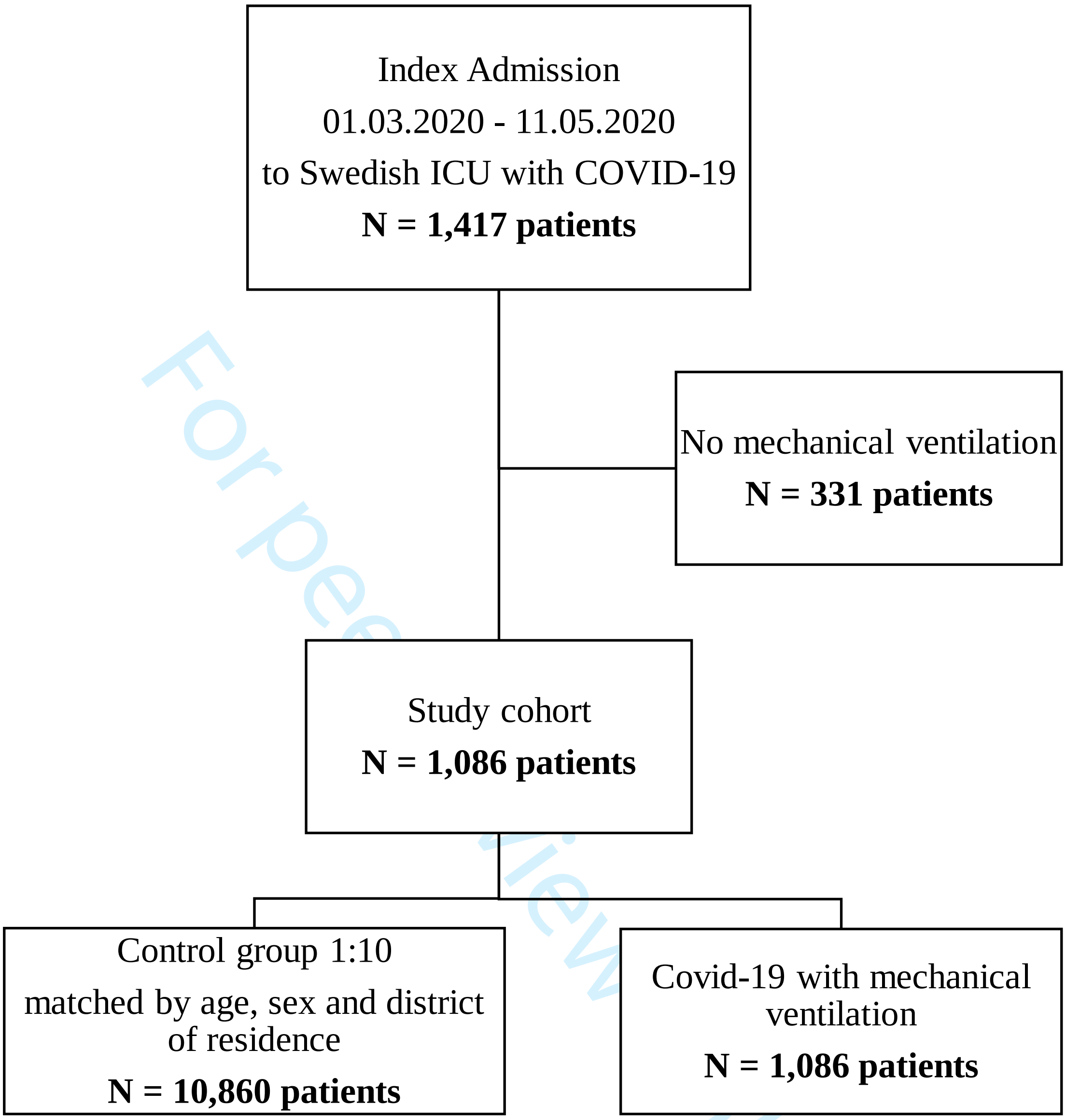

For peer review only - http://bmjopen.bmj.com/site/about/guidelines.xhtml

1. Ludvigsson JF, Andersson E, Ekbom A, Feychting M, Kim JL, Reuterwall C, Heurgren M and Olausson PO. External review and validation of the Swedish national inpatient register. *BMC Public Health*. 2011;11:450.

For peer review only

For peer review only - http://bmjopen.bmj.com/site/about/guidelines.xhtml

STROBE Statement—Checklist of items that should be included in reports of ***case-control studies***

	Item No	Recommendation	Page No
**Title and abstract**	1	(*a*) Indicate the study's design with a commonly used term in the title or the abstract	2
		(*b*) Provide in the abstract an informative and balanced summary of what was done and what was found	2
**Introduction**			
Background/rationale	2	Explain the scientific background and rationale for the investigation being reported	5
Objectives	3	State specific objectives, including any prespecified hypotheses	6
**Methods**			
Study design	4	Present key elements of study design early in the paper	6
Setting	5	Describe the setting, locations, and relevant dates, including periods of recruitment, exposure, follow-up, and data collection	6
Participants	6	(*a*) Give the eligibility criteria, and the sources and methods of case ascertainment and control selection. Give the rationale for the choice of cases and controls	6
		(*b*) For matched studies, give matching criteria and the number of controls per case	6
Variables	7	Clearly define all outcomes, exposures, predictors, potential confounders, and effect modifiers. Give diagnostic criteria, if applicable	7,8
Data sources/ measurement	8*	For each variable of interest, give sources of data and details of methods of assessment (measurement). Describe comparability of assessment methods if there is more than one group	7
Bias	9	Describe any efforts to address potential sources of bias	9
Study size	10	Explain how the study size was arrived at	6
Quantitative variables	11	Explain how quantitative variables were handled in the analyses. If applicable, describe which groupings were chosen and why	8
Statistical methods	12	(*a*) Describe all statistical methods, including those used to control for confounding	8
		(*b*) Describe any methods used to examine subgroups and interactions	8
		(*c*) Explain how missing data were addressed	8
		(*d*) If applicable, explain how matching of cases and controls was addressed	8
		(*e*) Describe any sensitivity analyses	
**Results**			
Participants	13*	(a) Report numbers of individuals at each stage of study—eg numbers potentially eligible, examined for eligibility, confirmed eligible, included in the study, completing follow-up, and analysed	Suppl Figure S2
		(b) Give reasons for non-participation at each stage	Suppl Figure S2
		(c) Consider use of a flow diagram	Suppl Figure S2
Descriptive data	14*	(a) Give characteristics of study participants (eg demographic, clinical, social) and information on exposures and potential confounders	9

For peer review only - http://bmjopen.bmj.com/site/about/guidelines.xhtml

		(b) Indicate number of participants with missing data for each variable of interest	Etable2
Outcome data	15*	Report numbers in each exposure category, or summary measures of exposure	Table 2

For peer review only

Main results	16	(*a*) Give unadjusted estimates and, if applicable, confounder-adjusted estimates and their precision (eg, 95% confidence interval). Make clear which confounders were adjusted for and why they were included	Table 3
		(*b*) Report category boundaries when continuous variables were categorized	n.a
		(*c*) If relevant, consider translating estimates of relative risk into absolute risk for a meaningful time period	n.a
Other analyses	17	Report other analyses done—eg analyses of subgroups and interactions, and sensitivity analyses	10
**Discussion**			
Key results	18	Summarise key results with reference to study objectives	13
Limitations	19	Discuss limitations of the study, taking into account sources of potential bias or imprecision. Discuss both direction and magnitude of any potential bias	15
Interpretation	20	Give a cautious overall interpretation of results considering objectives, limitations, multiplicity of analyses, results from similar studies, and other relevant evidence	16
Generalisability	21	Discuss the generalisability (external validity) of the study results	15
**Other information**			
Funding	22	Give the source of funding and the role of the funders for the present study and, if applicable, for the original study on which the present article is based	17

*Give information separately for cases and controls.

Note: An Explanation and Elaboration article discusses each checklist item and gives methodological background and published examples of transparent reporting. The STROBE checklist is best used in conjunction with this article (freely available on the Web sites of PLoS Medicine at http://www.plosmedicine.org/, Annals of Internal Medicine at http://www.annals.org/, and Epidemiology at http://www.epidem.com/). Information on the STROBE Initiative is available at http://www.strobe-statement.org.

For peer review only
